# Activated tissue resident memory T-cells (CD8+CD103+CD39+) uniquely predict survival in left sided “immune-hot” colorectal cancers

**DOI:** 10.3389/fimmu.2023.1057292

**Published:** 2023-05-11

**Authors:** Shahd Talhouni, Wakkas Fadhil, Nigel P. Mongan, Lara Field, Kelly Hunter, Sogand Makhsous, Alexandre Maciel-Guerra, Nayandeep Kaur, Ausrine Nestarenkaite, Arvydas Laurinavicius, Benjamin E. Willcox, Tania Dottorini, Ian Spendlove, Andrew M. Jackson, Mohammad Ilyas, Judith M. Ramage

**Affiliations:** ^1^ Cancer Immunology Group, School of Medicine, University of Nottingham Biodiscovery Institute, Nottingham, United Kingdom; ^2^ Faculty of Pharmacy, Al-Zaytoonah University of Jordan, Amman, Jordan; ^3^ Academic Unit of Translational Medical Sciences, School of Medicine, Queens Medical Centre, University of Nottingham, Nottingham, United Kingdom; ^4^ School of Veterinary Medicine and Sciences, University of Nottingham Biodiscovery Institute, Nottingham, United Kingdom; ^5^ Department of Pharmacology, Weill Cornell Medicine, New York, NY, United States; ^6^ Birmingham Tissue Analytics, College of Medical and Dental Sciences, University of Birmingham, Birmingham, United Kingdom; ^7^ School of Veterinary Medicine and Science, University of Nottingham, Sutton Bonington, United Kingdom; ^8^ Faculty of Medicine, Institute of Biomedical Sciences, Vilnius University, Vilnius, Lithuania; ^9^ Institute of Immunology and Immunotherapy, University of Birmingham, Birmingham, United Kingdom; ^10^ Host-Tumour Interactions Group, School of Medicine, University of Nottingham Biodiscovery Institute, Nottingham, United Kingdom

**Keywords:** colorectal cancer, T-cells, multiplex IHC/IF, tissue resident T cells, immune microenvironment, CD8 T-cells, cancer

## Abstract

**Introduction:**

Characterization of the tumour immune infiltrate (notably CD8+ T-cells) has strong predictive survival value for cancer patients. Quantification of CD8 T-cells alone cannot determine antigenic experience, as not all infiltrating T-cells recognize tumour antigens. Activated tumour-specific tissue resident memory CD8 T-cells (T_RM_) can be defined by the co-express of CD103, CD39 and CD8. We investigated the hypothesis that the abundance and localization of T_RM_ provides a higher-resolution route to patient stratification.

**Methods:**

A comprehensive series of 1000 colorectal cancer (CRC) were arrayed on a tissue microarray, with representative cores from three tumour locations and the adjacent normal mucosa. Using multiplex immunohistochemistry we quantified and determined the localization of T_RM_.

**Results:**

Across all patients, activated T_RM_ were an independent predictor of survival, and superior to CD8 alone. Patients with the best survival had immune-hot tumours heavily infiltrated throughout with activated T_RM_. Interestingly, differences between right- and left-sided tumours were apparent. In left-sided CRC, only the presence of activated T_RM_ (and not CD8 alone) was prognostically significant. Patients with low numbers of activated T_RM_ cells had a poor prognosis even with high CD8 T-cell infiltration. In contrast, in right-sided CRC, high CD8 T-cell infiltration with low numbers of activated T_RM_ was a good prognosis.

**Conclusion:**

The presence of high intra-tumoural CD8 T-cells alone is not a predictor of survival in left-sided CRC and potentially risks under treatment of patients. Measuring both high tumour-associated T_RM_ and total CD8 T-cells in left-sided disease has the potential to minimize current under-treatment of patients. The challenge will be to design immunotherapies, for left-sided CRC patients with high CD8 T-cells and low activate T_RM,_that result in effective immune responses and thereby improve patient survival.

## Introduction

Colorectal cancers (CRC) have traditionally been grouped together as one disease due to the anatomic continuity of the colon into the rectum. However, right and left colon differ in terms of their embryonic origin, vascular and nervous supplies, and gut flora (1). Increasing evidence has pointed to the location of the tumour affecting cancer pathology, progression and prognosis (2, 3), and ultimately patient’s response to different cancer treatments (4, 5). This in part may be due to the molecular variations and difference in mutational profiles between right and left-sided colon cancer (3, 6, 7). There is, however, increasing recognition in the literature that the differences in CRC may go beyond currently defined molecular subtypes (7) and that for cancer treatment right and left-sided colon cancer should be treated as separate diseases (3).

It is generally accepted that tumour progression may be influenced by non-malignant cells found in the tumour environment, especially the immune cells (8). T-cell infiltration has been shown to be superior to tumour staging as a prognostic factor (9, 10). Furthermore, responses to immune checkpoint blockade have been shown to be associated with patients having a pre-existing anti-tumour T-cell repertoire (11, 12). There is a strong correlation between the mutational load of a cancer type, the presence of neoepitopes and the response to immune check point blockade (13–15). Currently, only CRC patients with microsatellite instability (MSI), arising through loss of DNA mismatch repair function, are treated with checkpoint inhibitors (14). MSI is predominately found in right-sided colon cancers and tumours with MSI have been shown to have high T-cell infiltration (16, 17). Reflecting this, higher CD8 gene expression has also been demonstrated in right-sided colon cancer compared to the left-side (18).

Based on immune infiltration, tumours have been classified as: “immune-hot” (heavily infiltrated by lymphocytes); “immune-cold” (low levels of lymphocyte infiltration) (19). Since “immune-hot” tumours generally have a better prognosis than immune-cold tumours, this would imply that these immune cells – especially the CD8 T-cells – are activated and have anti-tumour activity. However, it has been shown that some tumour infiltrating T-cells may be inactive bystander cells which recognize viral antigens rather than cancer antigens (20).

Discriminating tumour-specific T-cells from bystander T-cells using markers of activation could aid in defining their role in the tumour microenvironment and patient outcome. One possible marker of activated CD8 T-cells is the co-expression of CD39 and CD103 (21). CD39 is an ectonucleotidase present on activated CD4 and CD8 T-cells following TCR stimulation (22). CD8^+^CD103^+^ have been defined as a tissue resident memory T-cell (T_RM_) signature (23). T_RM_ have been described as T-cells that no-longer circulate but develop and reside in the peripheral tissues as part of the memory response (24). CD103 (integrin alpha E), binds to E-cadherin and is important in retaining T_RM_ in the peripheral tissues (25).

The presence of CD103^+^ TIL in ovarian (26, 27), breast (26) lung (23, 28) and head and neck cancers (21) has shown a stronger correlation with survival than canonical T-cell markers such as CD3 or CD8. However, in a study in head and neck cancer, Duhen et al. (21) demonstrated that not all CD8^+^CD103^+^ T-cells were tumour-specific, and that some were the result of bystander recruitment. An increase in 4-1BB and Ki-67 expression on CD8^+^CD103^+^CD39^+^ T-cells lead the authors to hypothesize that these cells had recently encountered cognate antigen and were proliferating in the tumour. Moreover, these cells expressed granzyme and were cytotoxic. These tumour specific T-cells had an oligoclonal T-cell receptor (TCR) repertoire (21), and were associated with improved survival. Similarly, T-cells recognizing neoepitopes isolated from CRC patients expressed CD103 and CD39 (29), whereas bystander T_RM_ that were specific for viral antigens and not tumour antigens had diverse phenotypes that lacked CD39 expression (20). There is therefore substantial evidence that co-expression of CD103/CD39 can be used as a marker of activated T_RM_ in the tumour environment. Though these studies investigated tumour infiltrating lymphocytes they did not analyze the cells *in situ*. We hypothesized that intra-tumoural activated T_RM_ confer a survival advantage (by virtue of their tumour-specificity) in patients with CRC. Using multiplex immunohistochemistry, on a tissue microarray (TMA) containing cores from 1000 CRC patients, we quantified the activated and non-activated intra-tumoural CD8 T-cells in 891 cases. This study demonstrated that while high numbers of activated T_RM_ is a good predictor of survival in the overall cohort, there is a difference in the size of the effect depending on tumour location. In left-sided colon cancer high numbers of activated T_RM_ was the sole marker of enhanced survival irrespective of total CD8 T-cell infiltration. In contrast, in the right-sided colon cancer, both high numbers of activated T_RM_ and high total CD8 T-cell infiltration (even with low numbers of activated T_RM_) predicted good survival. This suggests that there is a fundamental difference in biology between right and left-sided colon cancers.

## Materials and methods

### Patient cohorts

Cohort 1 consisted of a consecutive series of 1000 CRC patients presenting at Nottingham University Hospitals NHS trust between 2008 and 2012 (53.6 months mean follow up). Cancer specific survival was measured from the date of primary surgical treatment to time of death due to cancer. Ethical approval was obtained for the study (reference no. 05/Q1605/66). The median age was 69 (range 16-94) with TMN stages: 16% stage I, 40% stage II, 32% stage III and 12% stage IV. For clinicopathological details refer to **Table 1**; **Supplementary Table 1**. Cohort 2 was obtained from the TCGA database containing mRNA sequencing data and clinicopathological characteristics (**Table 1**) of 515 colon adenocarcinomas (TCGA-COAD) (Cancer genome Atlas).

**Table 1:** Clinicopathological Characteristics of patient Cohorts 1 and 2.

Clinicopathological parameters		Cohort 1 All patients N (%)	Cohort 1 Left-sided CRC N (%)	Cohort 1 Right-sided CRC N (%)	Cohort 2 All patients N (%)	Cohort 2 Left-sided CRC N (%)	Cohort 2 Right-sided CRC N (%)
**Sex**	Male	568 (56.8%)	212 (58.4%)	236 (51.2%)	234 (52%)	65 (51.2%)	115 (52.8%)
	Female	432 (43.2%)	151 (41.6%)	225 (48.8%)	216 (48%)	62 (48.8%)	103 (47.2%)
	Overall	1000	363	461	450	127	218
**Age**	¾69	508 (50.8%)	196 (54%)	204 (44.3%)	240 (53.3%)	76 (59.8%)	104 (47.7%)
	>69	492 (49.2%)	167 (46%)	257 (55.7%)	210 (46.7%)	51 (40.2%)	114 (52.3%)
	Overall	1000	363	461	450	127	218
**Site of Primary tumour**	Right colon	461 (46.1%)	N/A	N/A	218 (48.4%)	N/A	N/A
	Left colon	363 (36.3%)	N/A	N/A	127 (28.25)	N/A	N/A
	Rectal	147 (14.7%)	N/A	N/A	1 (0.25)	N/A	N/A
	Unknown	29 (2.9%)	N/A	N/A	104 (23.15)	N/A	N/A
	Overall	1000	N/A	N/A	450	N/A	N/A
**N-regional** **Lymph nodes**	N0	570 (58.5%)	197 (56.3%)	274 (59.6%)	265 (58.9)	68 (53.5%)	133 (61%)
	N1	243 (24.9%)	106 (30.3%)	96 (20.9%)	104 (23.1)	40 (31.5%)	47 (21.6%)
	N2	162 (16.6%)	47 (13.4%)	90 (19.6%)	81 (18%)	19 (15%)	38 (17.4%)
	Overall	975	350	460	450	127	218
**Metastases** **(M)**	M0 (no distant metastasis)	881 (88.1%)	315 (86.8%)	406 (88.1%)	331(75.2%)	92 (73%)	159 (74%)
	M1 (Distant Metastasis)	119 (11.9%)	48 (13.2%)	55 (11.9%)	63 (14.3%)	23 (18.3%)	26 (12.1%)
	MX (unknown)				46 (10.5%)	11 (8.7%)	30 (13.9%)
	Overall	1000	363	461	440	126	215
**T stage**	T1	74 (7.4%)	35 (9.6%)	19 (4.1%)	11 (2.5%)	4 (3.1%)	5 (2.3%)
	T2	106 (10.6%)	31 (8.5%)	34 (7.4%)	77 (17.1%)	24 (18.9%)	35 (16.1%)
	T3	526 (52.6%)	190 (52.3%)	251 (54.4%)	308 (68.6%)	87 (68.5%)	143 (65.9%)
	T4	294 (29.4%)	107 (29.5%)	157 (34.1%)	53 (11.8%)	12 (9.4%)	34 (15.7%)
	overall	1000	363	461	449	127	217
**TNM stage**	I	161 (16.1%)	60 (16.5%)	47 (10.2%)	Not reported	Not reported	Not reported
	II	402 (40.2%)	138 (38%)	215 (46.6%)			
	III	319 (31.9%)	117 (32.2%)	145 (31.5%			
	IV	118 (11.8%)	48 (13.2%)	54 (11.7%)			
	Overall	1000	363	461			
**Vascular** **invasion**	Absent	502 (50.9%)	179 (50.1%)	228 (49.7%)	Not reported	Not reported	Not reported
	Present	483 (49%)	178 (49.9%)	231 (50.3%)			
	Overall	985	357	459			
**Microsatellite** **Status**	MSS	818 (83.6%)	336 (94.1%)	318 (70.4%)	Not reported	Not reported	Not reported
	MSI	160 (16.4%)	21 (5.9%)	134 (29.6%)			
	Overall	978	357	452			
**Treatment type**	Pharmaceutical	Not reported	Not reported	Not reported	231 (51.3%)	69 (54.3%)	110 (50.5%)
	Radiation				219 (48.7%)	58 (45.7%)	108 (49.5%)
	Overall				450	127	218

NA, not applicable.

### Tissue microarrays and immunohistochemistry

All tumours were formalin-fixed and processed into paraffin blocks. The histology of all cases was reviewed, appropriate donor blocks selected and marked to enable sampling of tumours from the luminal surface, center, advancing edge and adjacent normal mucosa. Tissue cores (0.6mm) were obtained from representative tumour regions of each donor block and arrayed into new recipient paraffin block using a tissue microarrayer (Beecher Instruments). Primary antibodies concentrations were optimized using chromogenic immunohistochemistry (30).

### Multiplex immunohistochemistry

Multiplex immunohistochemistry was performed using the Opal 4-colour manual IHC kit (Akoya Bioscience) for simultaneous detection of CD8, CD103 and CD39 on a single TMA section. Extensive optimization achieved fluorescence staining with optimal intensity and no bleed-through into other channels. Slides were deparaffinized in xylene and rehydrated in three baths of 100%, 90%, and 70% ethanol. Antigen retrieval was performed with EDTA (pH:9, 35 minutes, 100°C). TMA sections were incubated overnight (4°C) with anti-CD39 [Abcam, EPR20627, 1:300 in antibody diluent/blocking solution], washed with TBST buffer, incubated with anti-mouse-HRP conjugate, washed and incubated with Opal 570 (1:100) for 10 minutes. Antibody stripping was performed by microwaving (100W microwave: 100% for 45 seconds, 20% for 14 minutes) in antigen retrieval buffer (AR9, Akoya Bioscience) (31). Sides were cooled, washed and the procedure repeated with anti-CD103 antibody [Abcam: EPR4166(2) 1 hour, room temperature] paired with Opal 690 (1:100) and then with anti-CD8 [Agilent DAKO clone: C8/144b (M7103) 1 hour, room temperature] paired with Opal 520 (1:100). Slides were counterstained with Spectra DAPI, and coverslips were mounted with ProLong^®^ Diamond Antifade Mountant (Thermo Fisher Scientific). To obtain a spectral library anti-CD8 antibody was paired with each Opal fluorophore (without DAPI counterstain) as a singleplex.

### Image acquisition and scoring

**Figure 1:**
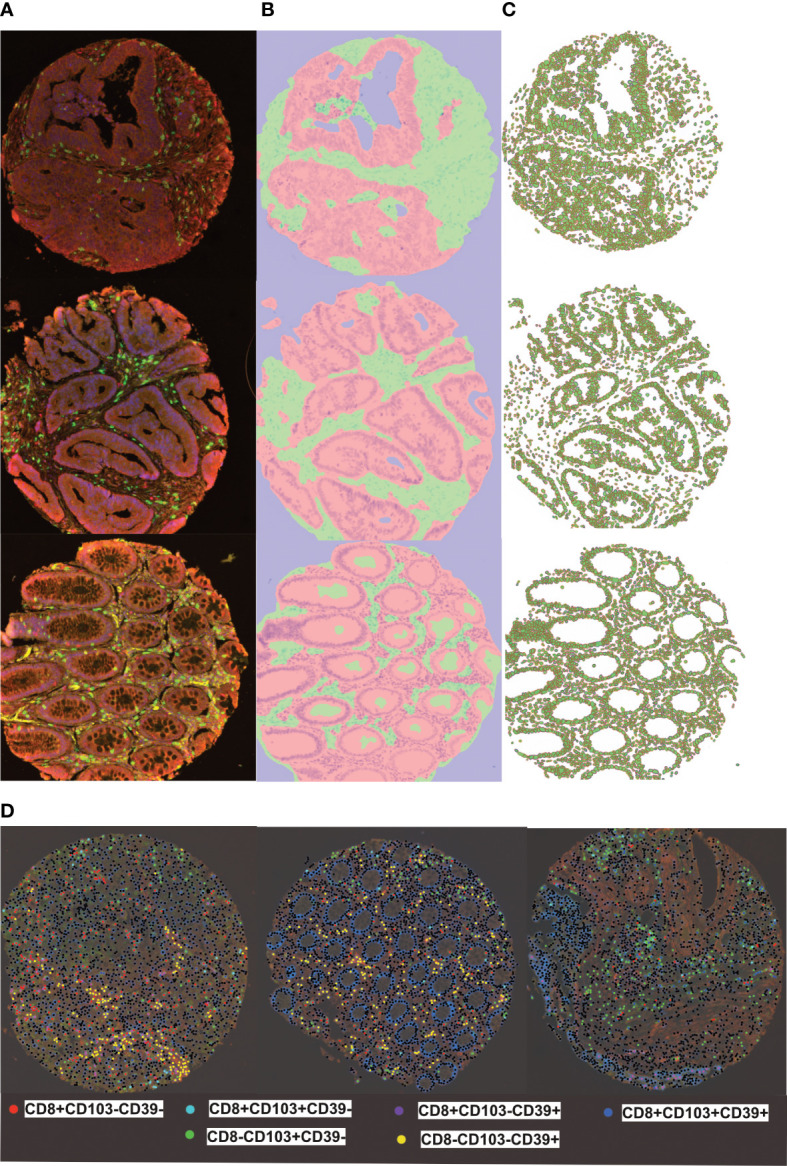
inform training stages for automated detection of tissues, cells and phenotyping individual cells **(A)** representive multiplex view of tumour cores with multi-stained cells, **(B)** Tissue segmentation of tumour epithelium (pink) and stroma (green) using pattern recognition **(C)** Cell segmentation was used to identify individual cell types **(D)** Training and phenotyping multi-stained and single stained cells was performed using a coloring- code method on infrom software. Black dots represent other cells.

TMA slides were scanned (Vectra 3, Akoya Biosciences) at 20 times resolution. The quality of the spectral library was evaluated by reviewing the unmixed images to confirm the absence of spectral overlap or bleed-through between channels. Machine learning (Inform® software, Akoya Biosciences**)** enabled quantification of cells in the stroma and epithelium. This consisted of training the software to recognize: stroma and epithelial cells; individual cells and phenotype different cells based on fluorescence (**Figure 1**; **Supplementary Figure 1**). Tissue segmentation involved training the program to detect differences between the stroma and epithelial cells based on tissue morphologies. DAPI staining allowed nuclei detection which was used to determine the cell segmentation. One schema was used to phenotype all the different subsets. They were trained as following: CD8+CD103+CD39+; CD8+CD103+CD39-; CD8+CD103-CD39+; CD8+CD103-CD39-; CD8-CD103+CD39-; CD8-CD103-CD39+ and other (any other cell). This allowed training to ensure that all double and triple positive cells were as phenotyped and that membrane staining was around the whole cell and not fluorescence from a neighbouring cell (**Supplementary Figure 2**). A separate schema was used to identify total CD8+ T-cells. All cores were visually inspected to determine agreement with machine learning: any that did not pass initial quality control were trained again (**Supplementary Figure 1**). The main reason for rejection was tissue segmentation (**Supplementary Figure 3** shows examples of failed and retrained images). Out of 4000 TMA cores, 3708 cores were accepted with the rest rejected due to loss of tissue or inaccurately trained after second training. The data from Inform® was merged and processed using phenoptrReports (Akoya Biosciences)

### Statistical analysis

Optimal cut-off points were determined using X-tile bioinformatics software (version 3.4.7). Statistical analysis was performed using the SPSS 21.0 software (SPSS, USA). Univariant and multivariant analyses were determined by chi-squared, log rank and Cox regression analysis, respectively. A p-value<0.05 was considered significant. GraphPad PRISM version 8 was used to compare the densities of different CD8+ T-cells. Statistical differences were assessed by two tailed paired T-test; One way Anova with Turkey’s multiple comparison test or Kruskal-Wallis with Dunn’s multiple comparison test.

### TCGA analysis

Using the medical reports from the TCGA-COAD database, patients were sorted according to primary tumour location: right-sided (228 patients) or left-sided (131 patients). HTSeq data were used as the gene expression count. Differentially expressed genes were determined in each sample group using Deseq2 (32). These groups were defined in terms of CD8 and CD103 expression: high (4^th^ Quartile) or low (1^st^ Quartile). Inclusion criteria for differentially expressed genes (DEG) was an FDR < 0.05, Log_2_ FC < -1 and Log_2_ FC < +1. FDR were analyzed with ClueGO (33), Cytoscape (34) and Webgestalt (http://www.webgestalt.org/) (35).

## Results

### Multiplex immunohistochemistry

**Figure 2:**
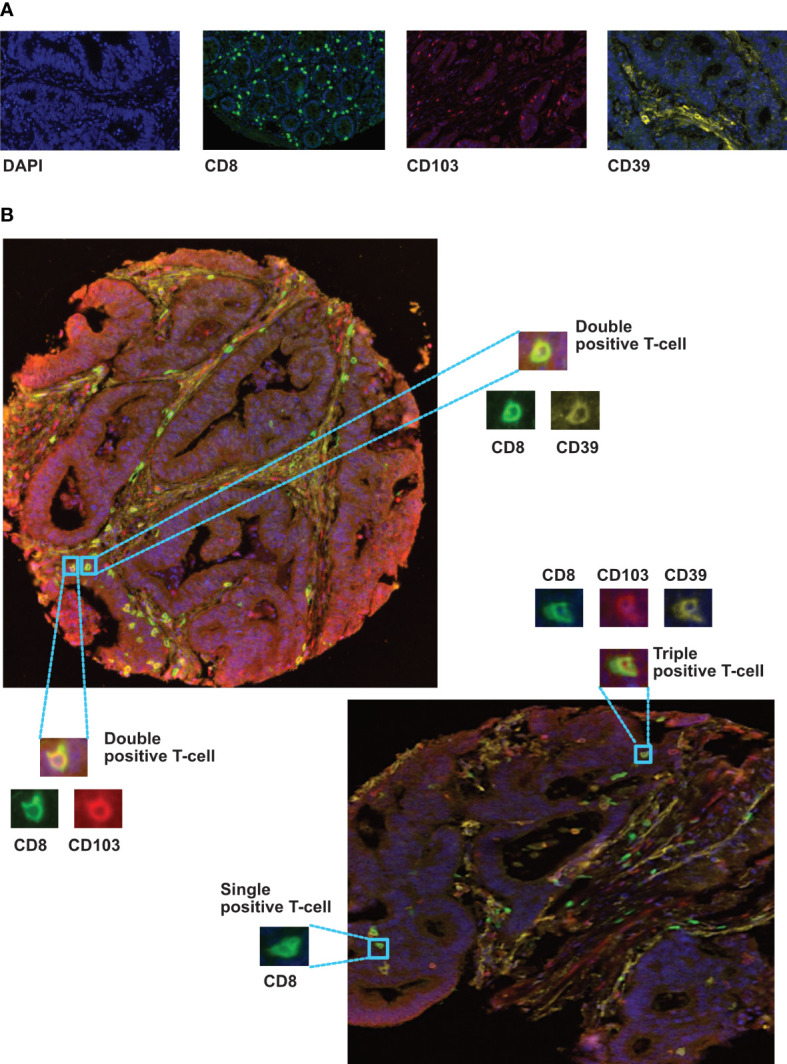
Immunolocalization of different CD8+ phenotypes in colorectal cancer tissues. **(A)** representive monoplex expression of CD8, CD103 and CD39 markers, and the tissue nuclear counterstain (DAPI) using fluorescence setting (Inserts x40). **(B)** Multiplex staining of different CRC TMA cores presenting triple, double and single stained cells with CD8 (green), CD103 (red) and CD39 (yellow) biomarkers. Training and phenotyping multi-stained and single stained cells were performed using coloring- code method on inform software.

Multiplex IHC was performed on a TMA containing tumour cores from 1000 colorectal cancer patients (cohort 1), each of which was represented by four cores from different tumour regions (see **Supplementary Figure 4**): luminal side; center of the tumour; invasive margin and adjacent pathologically normal. Representative examples of fluorescently labelled cells are shown in **Figure 2**. The following subsets were classified: CD8+CD103+CD39+ (activated T_RM_) CD8+CD103+CD39-, CD8+CD103-CD39+, CD8+CD103-CD39-, and total CD8+ T-cells. Cores from 891 patients remained available to analysis due to the loss of cores during processing and quality control for machine learning.

### Activated T_RM_ T-cells are an independent predictor of survival in CRC

**Figure 3:**
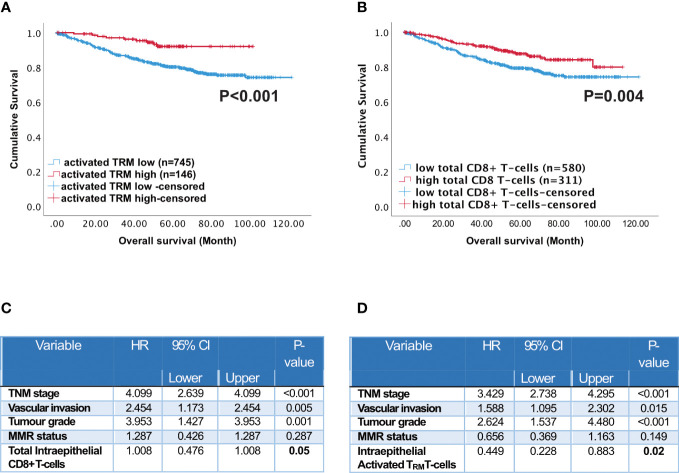
Prognostic impact of activated T_RM_ T-cells in CRC patients. Kaplan-Meier plots represent the probability of disease-specific survival for **(A)** Activated T_RM_ (CD8+CD103+CD39+) T-cells and **(B)** total CD8+ T-cells in the tumour epithelium. Cut-off points used to stratify CRC patients into high and low-density groups were determined using X-tile. The cut-off point for total CD8+ T cells was 222 cell/mm^2^ and for activated TRM cells was 74 cell/mm^2^. The log-rank test was used to compare curves, and p values <0.05 were considered statistically significant. **(C)** Multivariate analysis (Cox regression) of Intraepithelial total CD8 T cells with vascular invasion, TNM stage, tumour grade, microsatellite status and colorectal cancer-specific survival **(D)** Multivariate analysis (Cox regression) of Intraepithelial activated T_RM_ with vascular invasion, TNM stage, tumour grade, microsatellite status and colorectal cancer-specific survival.

Across the whole cohort, Kaplan Meier analysis indicated high numbers of activated T_RM_ in the epithelium (**Figure 3A**, p=0.004) and stroma (**Supplementary Figure 5**, p=0.006) were associated with increased survival. Patients with high numbers of activated T_RM_ in their tumours had a 10-year survival of 92% compared with 79% for total CD8+ T-cells (**Figures 3A, B**).

The abundant presence of high numbers of CD8 T-cells of any phenotype in the intra-epithelial region was significantly associated with disease-free survival (**Supplementary Figure 6** CD8+CD103+CD39- p<0.001; CD8+CD103-CD39+ p=0.017; CD8+CD103-CD39- p=0.006). In the stroma only high CD8 T-cells, activated T_RM_ and high CD8+CD103+CD39- T-cells were significantly associated with disease free survival (**Supplementary Figure 7**). Furthermore, all CD8 T-cell subsets were significantly correlated (p<0.05) with TNM stage, metastases, vascular invasion and microsatellite instability. High infiltration of activated T_RM_ was significantly associated with primary tumour location (see **Supplementary Table 2**). However, on their own neither MSI nor primary location predicted survival (**Supplementary Figure 8**). High numbers of intraepithelial activated T_RM_ was an independent prognostic factor (**Figure 3C** p=0.05). In agreement with the literature (36), high numbers of intraepithelial CD8+ T-cells was also an independent prognostic factor (**Figure 3D** p=0.05).

### Right-sided CRC patients with high CD8 and high CD103 gene expression have increased expression of genes involved in immune pathways

**Figure 4:**
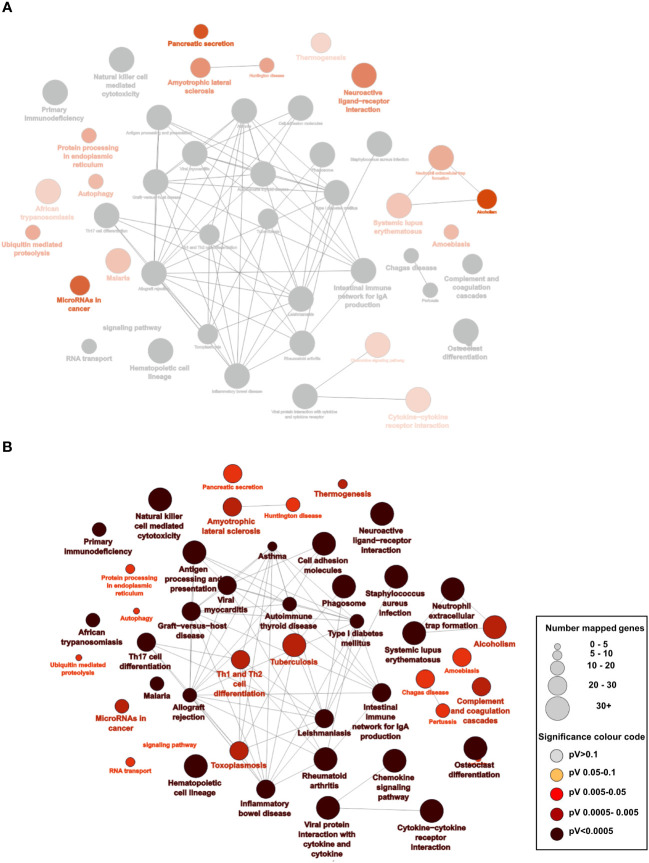
Overexpression of pathways predominately in right-sided CRC patients who have high CD8 and CD103 expression: **(A)** Analysis of the RNAseq data from the TCGA database of right- and left-sided CRC patients: 7245 differentially expressed genes (DEG) for right-sided CD8 high (4^th^ quartile) CD103 high (4^th^ quartile) patients compared with 877 DEG from left-sided CD8 high CD103 high CRC patients (p<0.05 and a logfold at least =1). There were predominately more significant enriched KEGG pathways on the right (shown in red) as opposed to left (where none were significant). The grey nodes represent overlapping pathways. The network of pathways was created using Cytoscape and ClueGo **(B)** Statistically enriched KEGG pathways with the larger the nodes representing more genes and the darker the red the more significant the pathway.

We utilized the data available in the cancer genome atlas (https://portal.gdc.cancer.gov/) to determine the differentially expressed genes (DEG) associated with high (4^th^ Quartile) and low (1^st^ Quartile) expression of CD8 and/or CD103 and the primary tumour location: right-sided (228 patients) or left-sided (131 patients). All groups were compared with CD8 low CD103 low from the same tumour side. Differential analysis identified 7272 DEG in the right-sided CD8 high CD103 high group. This contrast with only 877 DEG identified in the left-sided CD8 high CD103 high. The right- and left-sided CRC patients with CD8 high CD103 low expression had 260 DEG and 201 DEG respectively (**Supplementary Figures 9, 10** and **Supplementary Table 3**). Gene pathway comparison showed the right-sided CRC CD8 high CD103 high group had predominately more gene pathways than the comparable left-sided group (**Figures 4A, B**). Most of the pathways were associated with immunity. Pathways that are present in right but not left-sided CRC include: protein processing in ER; autophagy; ubiquitin mediated proteolysis; chemokine signaling pathways; cytokine-cytokine interaction and micro RNAs in cancer. There were limited pathways (**Supplementary Figure 10**) identified in the other groups and this included the high CD8 low CD103 expression group.

### Only activated T_RM_ T-cells predict survival in left-sided colon cancer

**Figure 5:**
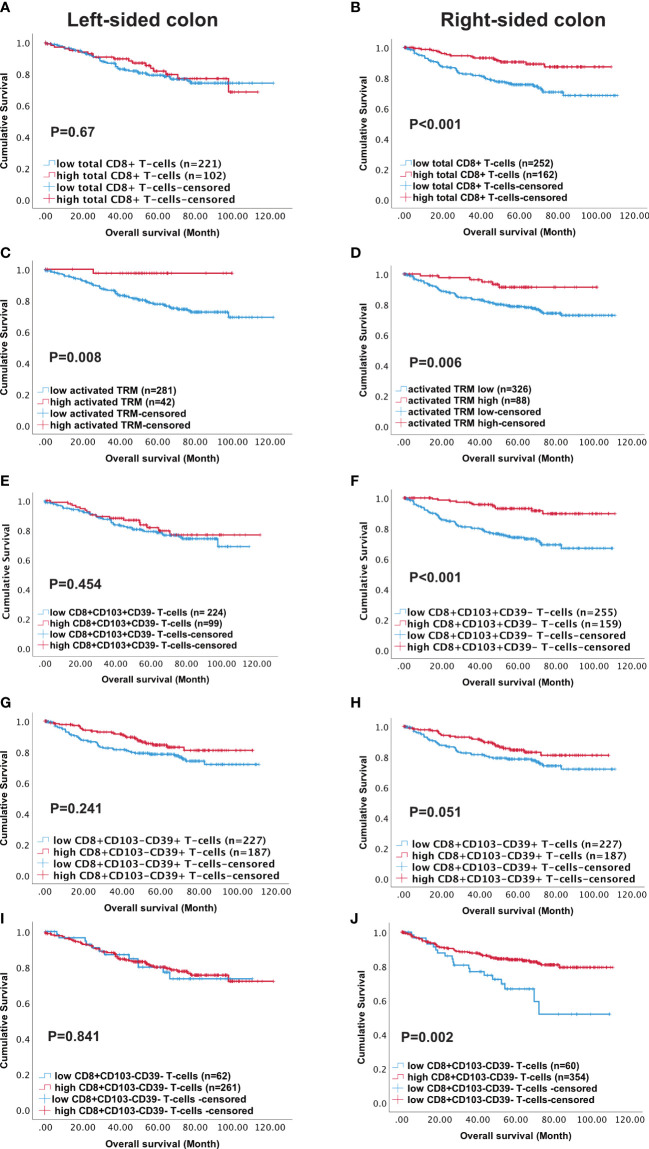
High density of activated T_RM_ T-cells predicts better survival in Left-sided colon cancer patients. Kaplan-Meier plots represent the probability of disease-specific survival for total CD8+ T-cells **(A, B)**, Activated T_RM_ (CD8+CD103+CD39+) T-cells **(C, D)**, CD8+CD103+CD39- T-cells **(E, F)**, CD8+CD103-CD39+ T cells **(G, H)** and CD8+CD103-CD39- T-cells **(I, J)** in the tumour epithelium of right-sided colon cancer and Left-sided colorectal cancer patients. Cut-off points used to stratify CRC patients into high and low-density groups were determined using X-tile. The cut-off points for total CD8+ T cells, activated T_RM_, CD8+CD103+CD39- T-cells, CD8+CD103-CD39+ T-cells and CD8+CD103-CD39- T-cells were 222, 74, 21, 19 and 7 cell/mm^2^, respectively. The log-rank test was used to compare curves, and p values <0.05 were considered statistically significant.

The TCGA analysis indicated the prevalence of immune pathways and DEG associated with high CD8 and high CD103 infiltration in right- versus left-sided CRC. We therefore analysed survival in our patient cohort in terms of tumour location. Although, high numbers of either total CD8+ T-cells or activated T_RM_ predicted survival this was not maintained in left-sided tumours. Indeed, although high infiltration of all immune phenotypes predicted survival in right-sided tumours, activated T_RM_ was the only phenotype that significantly predicted survival in left-sided disease (p=0.008) (**Figure 5**).

### Disparity between CD8 “immune-hot tumours” and T_RM_ T-cell infiltration

To assess if all “immune-hot” tumours contained elevated numbers of activated T_RM,_ heat maps were employed for all phenotypes. While the majority of patients with increased activated T_RM_ had corresponding elevations of total CD8 T-cells (**Supplementary Figure 11**), not all patients with high total CD8 T-cells had high levels of activated T_RM_. Neither the tumour location or MSI status was associated with any of the groups (**Supplementary Figure 9**).

### The absence of activated T_RM_ infiltration associates with poor prognosis irrespective of total CD8 infiltration

**Figure 6:**
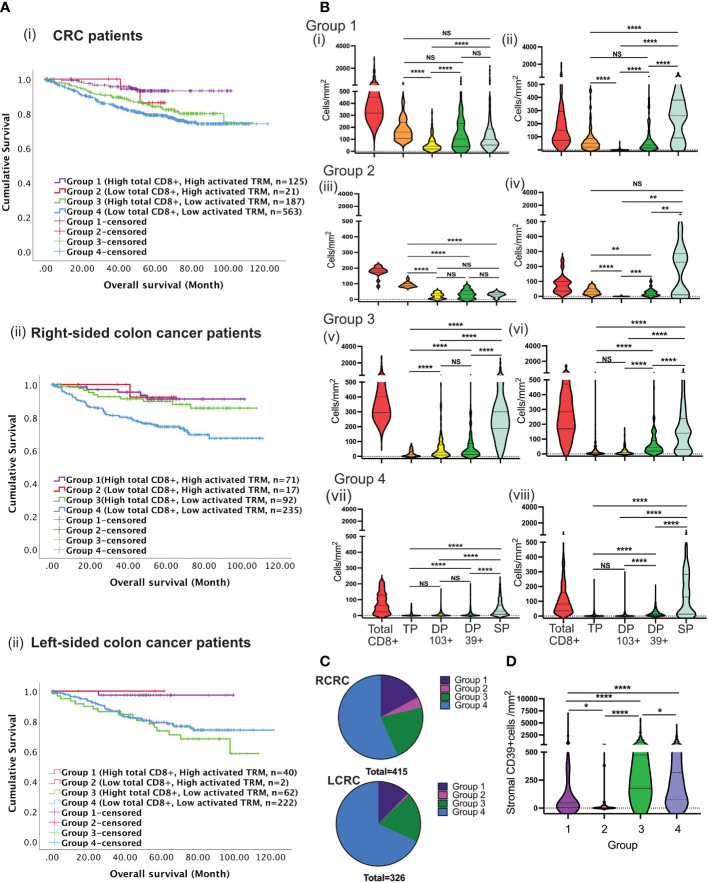
Tumours with high total CD8 T-cells and low activated TRM cells infiltration predict disease prognosis differently between RCRC and LCRC. **(A)** CRC patients were classified into four groups depending on total CD8 TILs, and activated TRM infiltration into the TME followed by the assessment of their correlation to survival using Kaplan Meier analysis for the entire (i) CRC cohort, (ii)Right sided and left sided colon cancer patients. **(B)** The violin plots represent the average densities of the respective CD8+ TIL subsets in the TME for each of the four groups using the Wilcoxon Test: Group 1((i) and (ii); Group 2 ((iii) and (iv)); Group 3 ((v) and (vi)); Group 4 ((vii) and (viii), The left column depicts intraepithelial CD8+ TIL ((i), (iii), (v) and (vii)) and the right column depicts stromal CD8+ TIL ((ii), (iv), (vii) and (viii) (TP: triple positive (CD8+CD103+CD39+), DP 103+ : double positive CD103(CD8+CD103+CD39-), DP 39+: double positive CD39+ (CD8+CD103-CD39+), SP: single positive (CD8+CD103-CD39+)) **(C)** The pie charts illustrate the numerical proportion of the four groups within RCRC (right-sided colon cancer patient) (n=415) and LCRC (left sided colon cancer patients) (n=326). **(D)** Comparison of average densities of CD39+ non-lymphoid cells in the stroma of group 1, group 2, group 3 and group 4 tumours. One way Anova/mixed effect analysis with Turkey correction for multiple comparison was used for statistical comparisons in B, while a Kruskal-wallis test with Dunn’s multiple comparison test was used in **(D)** p-values <0.05 were considered statistically significant. (ns if p > 0.05, *p ≤0.05, **p ≤ 0.01, ***p ≤ 0.001, ****p ≤ 0.0001).

Survival was studied in patients with high (“immune-hot”) or low (“immune-cold”) total CD8 T-cells and high or low numbers of activated T_RM_ in the tumour epithelium resulting in 4 groups of patients. Group-1 patients (high CD8 T-cell infiltration and high activated T_RM_) had the best survival overall **Figures 6A(i), B(i)** p=0.003). The major CD8+ subset in the tumours were activated T_RM_. Furthermore, there was greater infiltration of these cells in the epithelial than stroma (p<0.0001) **Figure 6B(i)**: such tumours could be conventionally classified as “immune-hot”.

Group-2 tumours lacked pronounced CD8+ T-cell infiltration but nevertheless exhibited high numbers of activated T_RM_ **Figures 6A(i), B(ii)**. These tumours displayed significantly greater CD8 infiltration than Group-4 (low total CD8 low activated T_RM_) (p<0.0001) **Figure 6B(iv)**. Survival in Group-2 patients was not significantly different to Group-1 indicating that the presence of activated T_RM_ (as opposed to total CD8) are important for survival.

Group-3 had high total CD8 T-cell infiltration but low numbers of activated T_RM_. Most cells were single positive (CD8+) with few CD8+CD103+ or CD8+CD39+ cells **Figure 6B(iii)**. They had a similar pattern of infiltration in the intraepithelial and stroma. However, despite having high total CD8+ T cells, patients in this group had poor survival. Lastly, group-4 displayed both low total CD8+ infiltration and low numbers of activated T_RM_ with corresponding poor survival **Figure 6B**. This group were conventional “immune-cold” tumours **Figure 6B(iv)**. Therefore, only patients with high numbers of CD8 T-cells and/or high numbers of activated T_RM_ had improved survival (truly hot tumours). Importantly, patients whose tumours exhibited high total CD8 T-cells in the absence of activated T_RM_ had poor survival **Figure 6A(i)**.

### Activated T_RM_ are required for better survival prognosis in left-sided but not right-sided colon cancer

Sub-classification of patients into right and left-sided disease revealed differential survival **Figures 6A(ii), (iii)**. For patients with left-sided tumours survival depended on infiltration by activated T_RM_. In contrast, for right-sided disease a group of patients exhibited good survival with high total CD8+ T-cells but low activated T_RM_ infiltration. There was no difference in overall number of infiltrating cells between right- and left-sided tumours in any group (data not shown). However, survival was poor in patients with left-sided disease (**Figure 6C**, Group 3).

### The presence of CD39+ on non-lymphocyte stromal cells was associated with poor survival

CD39 and CD73 ectonucleotidases operate in concert to produce immunosuppressive metabolite adenosine in tumour environments (37). We hypothesized that high levels of CD39+ cells in the non-lymphoid stroma cells (e.g., fibroblast) associated with poor survival. As shown in **Figure 6D** higher numbers of CD39+ cells were observed in group-3 and group-4 patients: those that lacked abundant activated T_RM_.

### Infiltration of total CD8 and activated T_RM_ T-cells consistent across the cores

**Figure 7:**
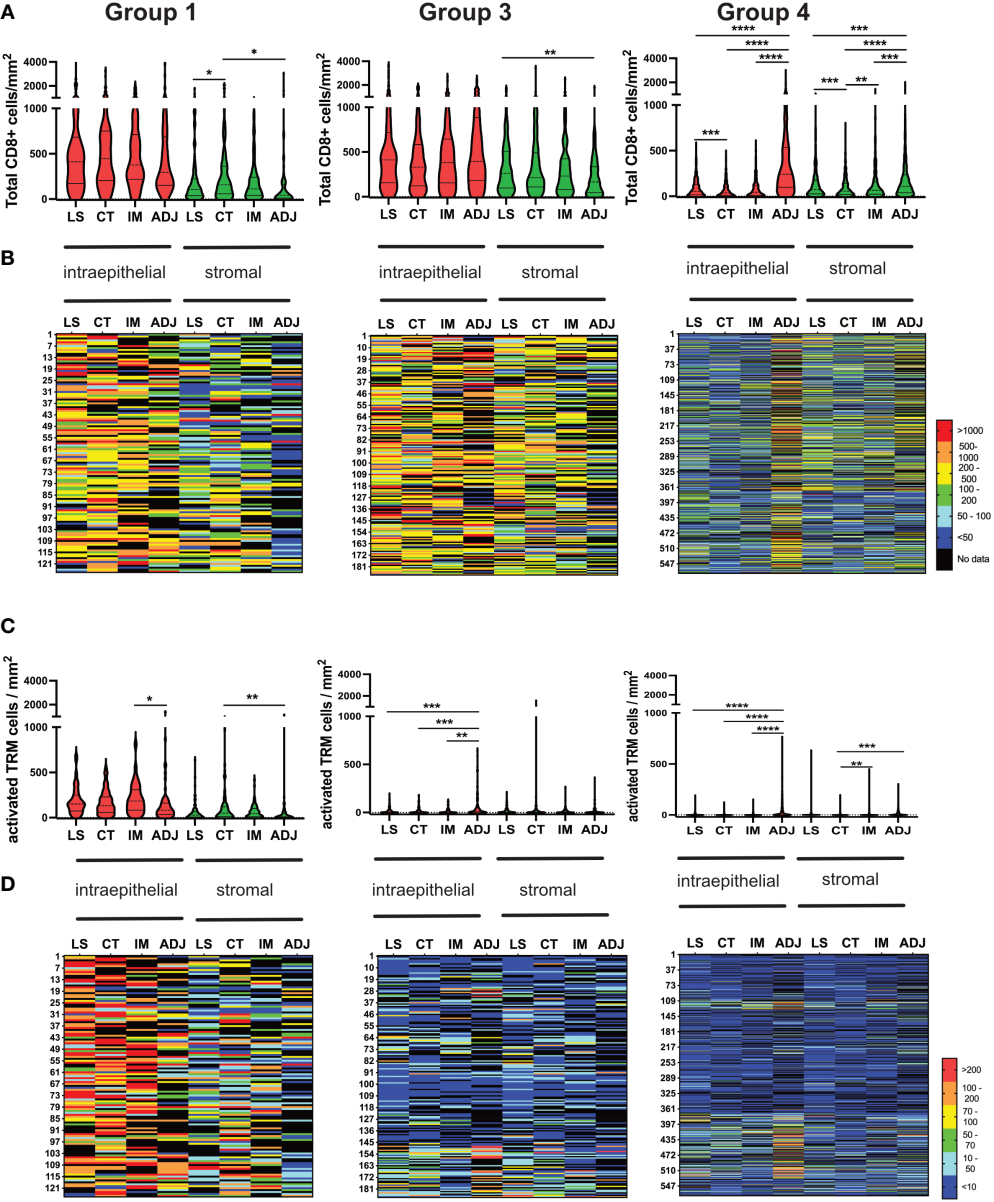
Consistent Infiltration of recently activated TRM cells across different tumour regions (LS, CT, IM and ADJ) within the four groups of CRC cohort. **(A)** The violin plots represent the median density of total CD8 TILs in the respective tumour region matched to the density of total CD8 TILs infiltrating the same tumour region in each patient mapped one below each other in the heat maps **(B)**. Group 1 and group 3 tumours had no significant difference in total CD8 TILs infiltration between LS, CT and IM. The normal adjacent tissue had significantly higher total CD8 TILs infiltration compared to other tumour regions in group 4 tumours (Cold tumours). **(C)** The violin plots represent the median density of TP TRM cells in the respective tumour region matched to the density of TILs infiltrating the same tumour region in each patient in the heat maps **(D)**. Group 1 tumours (hot tumours) had significantly higher TRM cell infiltration in the LS, CT and IM as compared to ADJ. Meanwhile, group 3 and group 4 tumours had significantly higher activated TRM cell infiltration in the AJD as compared to other regions of the tumour. Mixed effect analysis with Turkeys multiple comparison correction test was used to test for significance. P values <0.05 were considered statistically significant. (*p ≤0.05, **p ≤ 0.01,***p ≤ 0.001, ****p ≤ 0.0001) (LS, luminal side; CT, center of tumour; IM, invasive margin and ADJ, adjacent normal).

Colorectal tumours have been shown to exhibit higher levels of immune infiltrate at the invasive margins (38). However, in this study infiltration of both total CD8 and activated T_RM_ was consistent throughout the tumours (**Figure 7**). Group-1,” immune-hot tumours”, had high numbers of CD8+ T-cells throughout the tumour with lower numbers in adjacent normal tissue. These tumours also had corresponding elevated levels of activated T_RM_ even in the adjacent pathologically normal tissue. Group-3 tumours were characterized by high levels of CD8 T-cells in all cores with no significant difference in any location and limited activated T_RM_ irrespective of region. In contrast, Group 4 “immune-cold” had scant CD8 T-cells throughout, but in adjacent normal tissue these cells were as abundant as those observed in Group-1 and Group-3. This group also had limited infiltration with activated T_RM_.

## Discussion

Our study of T_RM_ cells within the tumour environment is the largest and most detailed study of its kind, having determined the *in-situ* localization of CD8 T-cell subsets in tissue from nearly 900 CRC patients. We addressed whether the presence of all CD8 T-cells within the tumour are beneficial for survival or whether activated T_RM_ are a better predictor of survival. Using multiplex IHC we established the prognostic significance of activated T_RM_ in CRC. The presence of activated T_RM_ independently predicted survival. Furthermore, the presence of high numbers of activated T_RM_ was a better overall predictor of survival than total CD8+ T-cells. Tumours with high numbers of activated T_RM_ were predominately “immune-hot” (tumours with high total CD8+ T-cell counts), and were highly infiltrated throughout (luminal side, center of the tumour and invasive margins) with both total CD8+ T-cells and activated T_RM_. While CD8+ T-cells alone did not predict survival in left-sided tumours, activated T_RM_ were uniquely able to predict survival. This contrasted with right-sided tumours where patients exhibiting high total CD8+ T-cells (even with low numbers of activated T_RM_) had good overall survival.

It has previously been shown that CD8+CD103+CD39+ T-cells identifies tumour reactive T-cells in both head and neck cancer (21) and breast cancer (39), and that their presence associates with survival. While those studies showed the functionality of the activated T_RM_, we focused on their survival advantage and location in tumours. Our findings that activated T_RM_ are an independent predictor would support the concept that these T-cells have been active in the tumour environment.

The immunoscore uses the presence of CD3 and CD8 T-cell in the invasive margins and center of the tumour to predict survival in CRC (40–42). As far as we are aware we are the first to show that CD8 alone does not predict survival in left-sided colon cancer and that T_RM_ uniquely predict survival. Other studies have shown an increase in CD8 gene expression in right-sided CRC and suggested that these patients have a better immune response (18). Indeed, TCGA analysis of right-sided CRC patients with high CD8 and high CD103 compared to the low CD8 low CD103 had significantly more immune pathway involvement than patients with left-sided tumours, suggesting greater immune responses in right-sided disease. This is the first time both high CD8 and high CD103 expression have been analysed together using data from the TCGA database for CRC and the results are consistent with our data showing that there are differences in immune responses between right and left-sided CRC.

The presence of high CD8 T-cells irrespective of subtype predicts survival in right-sided CRC. Intriguingly, “immune-hot” tumours with low numbers of activated T_RM_ had good survival, suggesting that although activated T_RM_ are important as predictors of survival there may be other markers of antigen specificity and activation that need to be defined for these patients. Why infiltration of high total CD8+ T-cells without T_RM_ is a good prognosis on right-sided but not left-sided colon cancer is unclear, however, other leukocytes within the tumours may be contributing factors. These could include T-regs and tumour associated macrophages. In a study in breast cancer, the presence of T-regs was responsible for the loss of prognostic significance of high numbers of T_RM_ (39).

The difference in survival relating to T-cell infiltration in the right versus the left CRC could be due to differences in somatic mutations and neoantigen generation. The mutations rates in *BRAF*, *POLE, POLD1* and *PIK3CA* genes associate with right-sided CRC. Patients with these mutations (43) have elevated expression of helper T-cells, class II-related genes, chemokines and inhibitory molecules. In contrast, *RAS* mutant (KRAS, NRAS) tumours (which are more frequent on the left) associate with poor immune infiltration, low inhibitory molecule expression (43) and recruitment of suppressive myeloid cells. Nevertheless, this does not explain why CRC patients with tumours heavily infiltrated with CD8 T-cells but lacking activated T_RM_ have good prognosis if their tumour is right-sided. It is not that the T_RM_ have not upregulated CD39 in these tumours, but that there are low levels of T_RM_: most cells are single positive CD8 T-cells. The literature would support the theory that CD8+CD103+CD39- T-cells are bystander without specificity for tumour antigens (20) while activated T_RM_ are tumour specific. Left-sided CRC generally exhibit lower mutational burden, and therefore may present less antigenic opportunity for antigen specific T_RM_. The association between the absence of activated T_RM_ with high levels of CD39+ stromal cells (e.g. fibroblasts) may reflect the role of CD39 in ATP depletion and suppression of T-cell activation and may consequently account for the lack of T_RM_ in these patients. Quantifying activated T_RM_ in CRC patients may therefore permit stratification of tumours with strong immunity genuinely focused on the tumour.

Bindea et al. (38) in a study of 107 patients, showed elevated total CD8+ cells in the invasive margins than the rest of the tumour. In contrast, we observed in 9-fold more patients that high total CD8+ T-cells were homogeneously distributed throughout the “immune-hot” tumours. Whereas in “immune-cold” tumours there were higher numbers of CD8+ T-cells present in pathological adjacent normal tissue than in the tumours. The difference could be due to their analysis of the whole cohort of CRC patients together compared to our analysis of patients with high and low total CD8 T-cell separately.

CD39 and CD103 upregulation on tumour infiltrating CD8 T-cells is due to TGFβ and chronic TCR stimulation (21). Once a cell is activated it displays markers of exhaustion. The expression of CD39 and TIM3 have been shown to discriminate exhausted cells from memory/effector T-cells (44). Expression of other markers of exhaustion such as PD-1 associate with terminal exhaustion (45). It is of note that in our study the “immune-hot” tumours had high levels of all CD8 phenotypes including both CD8+CD103+ and CD8+CD39+. This could suggest an active immune response in the tumour environment of these patients and therefore the reason activated T_RM_ herald survival is that they have previously responded to the tumour. It can be argued that patients who have previously mounted an immune response could continue to mount new T-cell responses.

Clinical use of immune checkpoint inhibitors has revolutionized the treatment of solid tumours. However, future work is needed to identify CRC patients suitable for immunotherapy and characterize repertoires of T-cells to be targeted, a point illustrated by studies showing the presence of intra-tumoural CD103+CD8+ T cells predicts responses to PDL-1 blockade (46, 47). We have already argued that activated T_RM_ have markers of exhaustion, and that the presence of T_RM_ in the tumour is indicative of active immunity. While there is evidence that exhausted T-cells can respond to checkpoint blockade (48, 49), there is also evidence that these cells are not readily reactivated (50). Indeed, recent studies show that new responses to checkpoint inhibitors involve CD8 T-cells actively replenished from outside the tumour (50) and not from the pre-existing repertoire of exhausted T-cells (51). Patients who have previously mounted an immune response could continue to mount new responses with appropriate immunotherapy. In this paper we have shown over 90% survival in the group of patients with high numbers of total CD8 and high numbers of activated T_RM_. Therefore, patients who may benefit from future immunotherapies may not be the ones with activated T_RM_ who already have generated a good immune prognosis but those patients who have shown the ability to recruit CD8 T-cells but have so far failed to convert this to a survival advantage: the left-sided colon cancer patients with high CD8 T-cells and low recently activated T_RM_. The challenge will be to generate activated tumour specific T_RM_ in these patients.

## Data availability statement

The raw data supporting the conclusions of this article will be made available by the authors, without undue reservation.

## Ethics statement

The studies involving human participants were reviewed and approved by Oxford Rec B, NHS (reference 05/Q1605/66). The patients/participants provided their written informed consent to participate in this study.

## Author contributions

Data acquisition: ST, NM, KH, SM, AN, AL. Analysis and interpretation of data: JR, ST, NM, LF, NK, IS, AJ, MI, AM-G, TD. Building the patient cohort: WF, MI, Conception/design of work: JR, AJ, ST. Oversight of entire project: JR. Manuscript preparation and review: JR, ST, AJ, MI, IS, BW. All authors contributed to the article and approved the submitted version.
