# Supplementary material for: Activated tissue resident memory T-cells (CD8+CD103+CD39+) uniquely predict survival in left sided “immune-hot” colorectal cancers
Source: Front Immunol. 2023 May 11;14:1057292. doi: 10.3389/fimmu.2023.1057292 (PMC10213916; doi:10.3389/fimmu.2023.1057292)
Supplement: Supplementary file 1 [file DataSheet_1.pdf]

Supplementary Table 1: – Summary of the clinicopathological characteristics of the patient cohort

Clinic-pathological parameters		N (%)	Mean (DSS) months	SE	95% confidence interval		Survival distribution (Log Rank- Mantel cox) of overall colorectal patients	
					Lower	Upper	Chi-Square	P value
**Gender**	**Male**	568 (56.8%)	99.9	1.86	92.9	100.2	1.51	0.219
	**Female**	432 (43.2%)	96.6	1.85	96.3	103.6		
	**Overall**	1000 (100%)	101.2	1.39	98.5	104		
**Age**	**(≤69)**	489	101.2	1.91	97.48	104.9	0.004	0.95
	**(>69)**	511	96.65	1.88	92.98	100.32		
	**Overall**	1000	101.2	1.39	98.51	103.9		
**N -Regional lymph nodes**	**N0 (no lymph node metastasis)**	570 (58.5%)	106.82	1.23	104.41	109.23	103.35	**<0.001**
	**N1 (Regional lymph node metastasis)**	405 (41.5%)	78.79	2.36	74.18	83.42		
	**Overall**	975 (100%)	96.26	1.313	93.69	98.83		
**Metastases (M)**	**M0 (no distant metastasis)**	881 (88.1%)	107.6	1.28	105.05	110.07	272.73	**<0.001**
	**M1 (Distant metastasis)**	119 (11.9%)	45.3	4.04	37.4	53.22		
	**Overall**	1000 (100%)	101.24	1.39	98.51	103.97		
**Site of primary tumour**	**Right colon**	461 (46.1%)	93.07	1.87	89.41	96.74	3.995	0.262
	**Left colon**	363 (36.3%)	99.53	2.33	94.95	104.12		
	**Rectal**	147 (14.7%)	98.02	2.76	92.61	103.44		
	**Unknown**	29 (2.9%)	103.82	5.08	93.85	113.8		
	**Overall**	100 (100%)	101.24	1.39	98.51	103.97		
**TNM stage**	**I**	161 (16.1%)	119.2	1.28	116.7	121.7	307.781	**<0.001**
	**II**	402 (40.2%)	103.7	1.41	100.9	106.5		
	**III**	319 (31.9%)	88.8	2.36	84.15	93.43		
	**IV**	118 (11.8)	45.4	4.08	37.43	53.45		
	**Overall**	1000 (100%)	101.24	1.39	98.51	103.9		
**Extramural vascular invasion**	**Absence**	502 (50.9%)	110.9	1.48	108.0	113.8	56.655	**<0.001**
	**Present**	483 (49%)	84.1	2.07	80.13	88.3		
	**Overall**	985 (100%)	101	1.404	98.32	103.8		
**Microsatellite status**	**Microsatellite stable (MSS)**	818	100.6	1.54	97.59	103.6	2.082	0.149
	**Microsatellite instable (MSI)**	160	95.07	2.66	89.85	100.2		
	**Overall**	978	101.5	1.398	98.75	104.2		

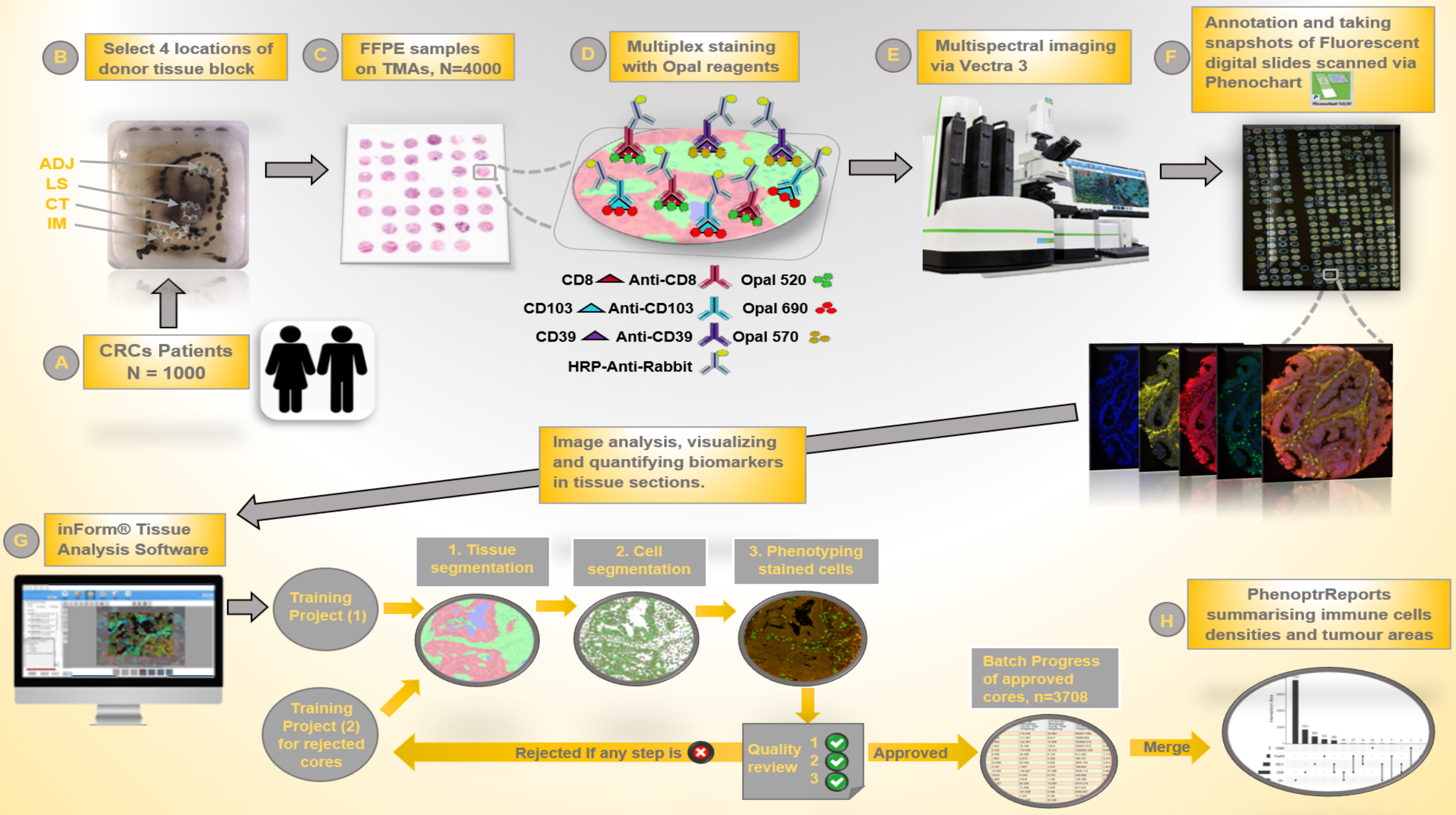
B
Select 4 locations of donor tissue block
C
FFPE samples on TMAs, N=4000
D
Multiplex staining with Opal reagents
E
Multispectral imaging via Vectra 3
F
Annotation and taking snapshots of Fluorescent digital slides scanned via Phenochart
ADJ
LS
CT
IM
CD8
Anti-CD8
Opal 520
CD103
Anti-CD103
Opal 690
CD39
Anti-CD39
Opal 570
HRP-Anti-Rabbit
A
CRCs Patients N = 1000
Image analysis, visualizing and quantifying biomarkers in tissue sections.
G
inForm® Tissue Analysis Software
1. Tissue segmentation
2. Cell segmentation
3. Phenotyping stained cells
Training Project (1)
Training Project (2) for rejected cores
H
PhenoptrReports summarising immune cells densities and tumour areas
Batch Progress of approved cores, n=3708
Rejected If any step is
Quality review
1
2
3
Approved
Merge

Supplementary figure 1: Diagram representing process of multiplex IHC and machine learning: (**A)** CRC primary tumours from 1000 patients (Males 57%,females 43%). **(B)** Samples extracted four different locations of donor tissue block. **(C)** TMA Construction consists of 4000 cores. **(D)** Multiplex staining with Opal 4-color IHC Kit anti-CD8, anti-CD39 and anti-CD103 antibodies were paired with Opal 520, 570 and 690, respectively. **(E)** Vectra 3 Imaging System used for multispectral imaging. **(F)** Phenochart™ viewer was used for annotation and taking snapshots of whole slides. **(G)** inForm® software was used to detect and quantify different phenotypes within the TMA core. InForm® training project enabled tissue and cell segmentation and phenotyping stained cells automatically. The quality of the project outcome was checked, and a new project was used to correct the rejected cores. **(H)** PhenotrReports calculated the densities of the stained cells and the tumour (epithelium and stroma) area

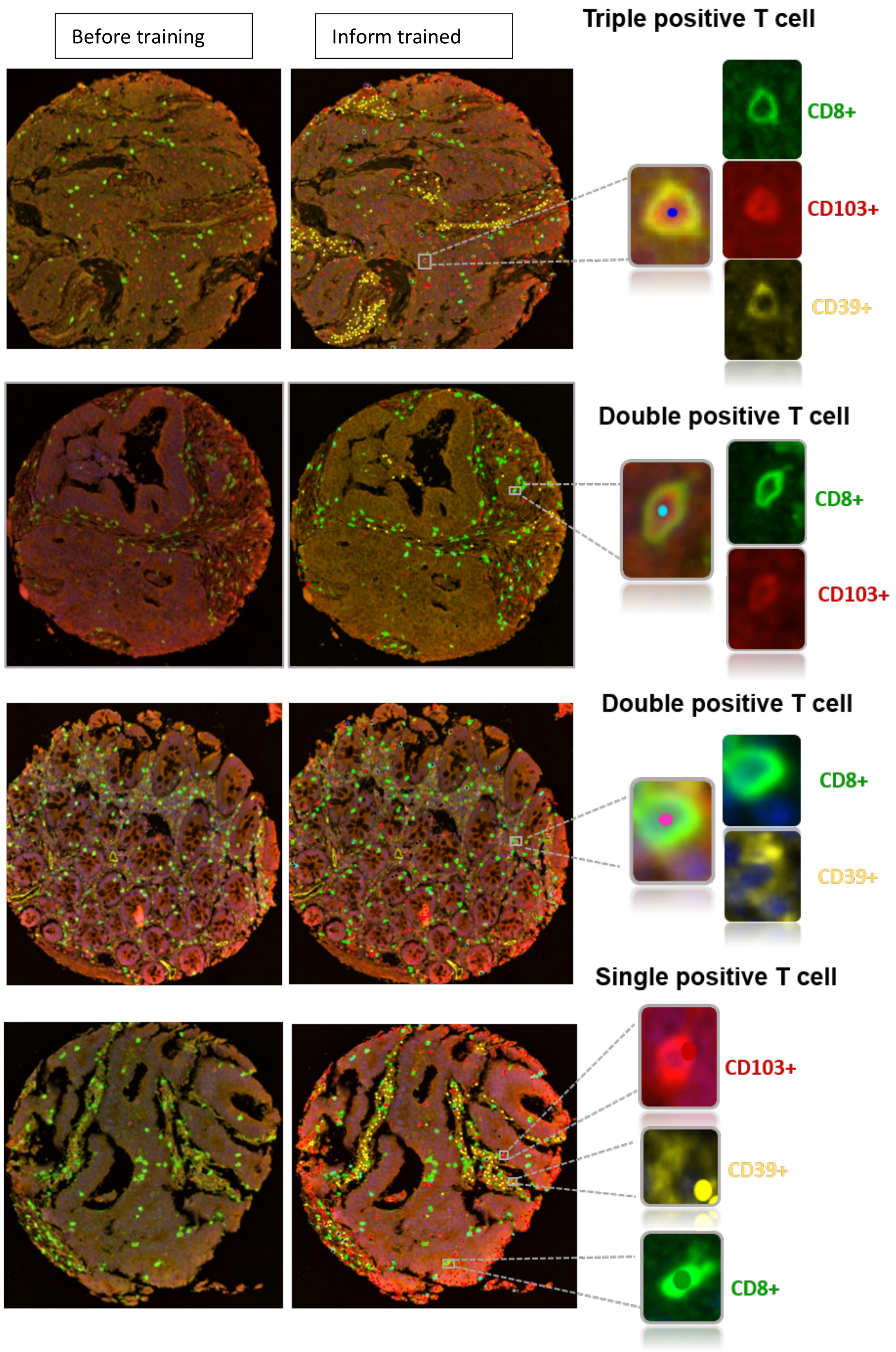

Supplementary Figure 2: Training and phenotyping multi-stained and single stained cells using colouring- code method on infrom software.

inForm® was trained to detect and quantify six different TILs phenotypes: CD8+CD103+CD39+(dark blue), CD8+CD103+CD39- (turquoise), CD8+CD39+(magenta), CD8+ (green), CD8-CD103+CD39- (red) and CD8-CD103-CD39+(yellow) TILs. This involved giving examples of cells positive for these phenotypes and identifying cells that were negative

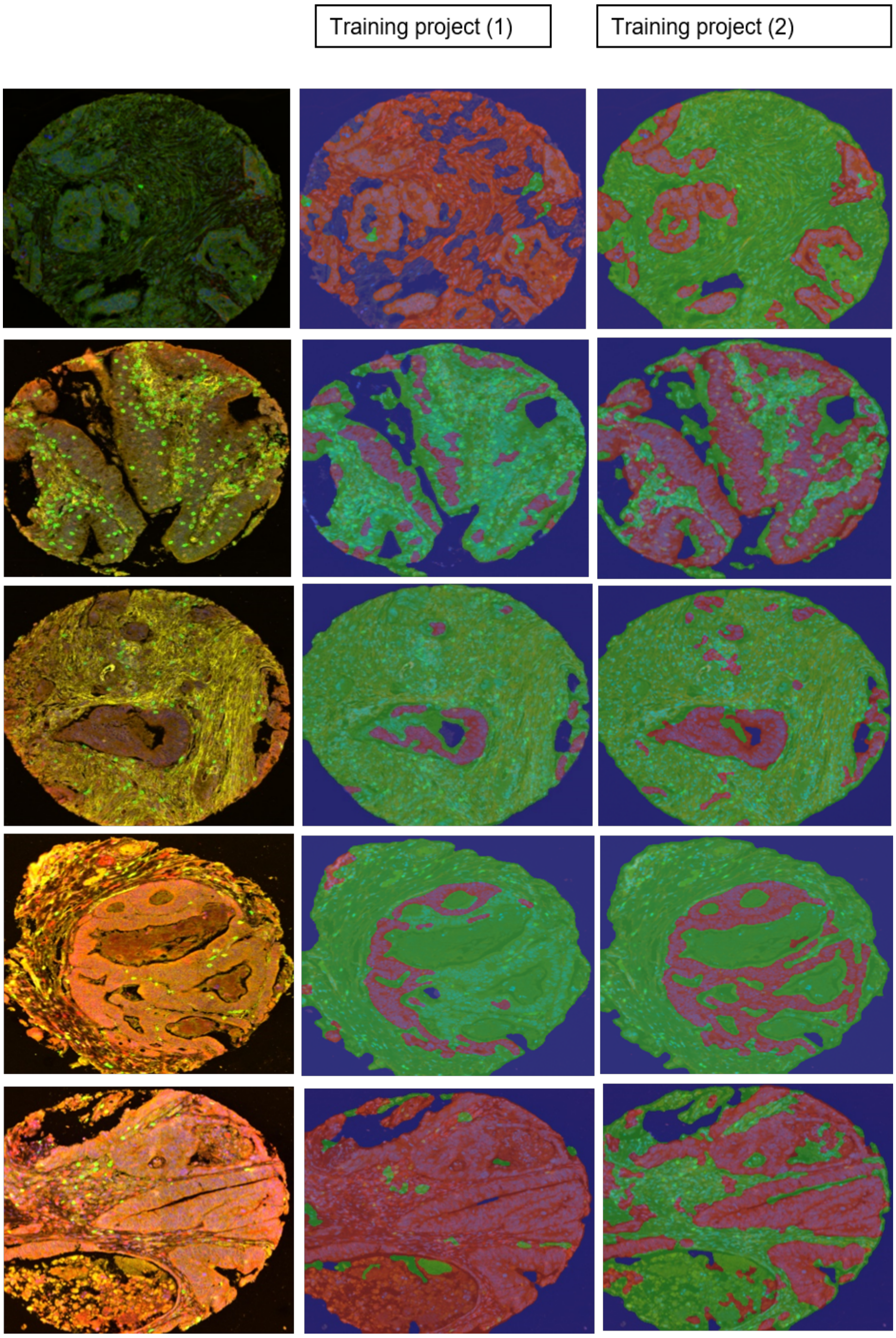

Supplementary figure 3: Examples of rejected cores from Project (1) and their corrected matches from Project (2).

The majority of the rejected cores showed inaccurate tissue segmentation from project (1). The rejected cores were corrected in project (2) to obtain better tissue segmentation.

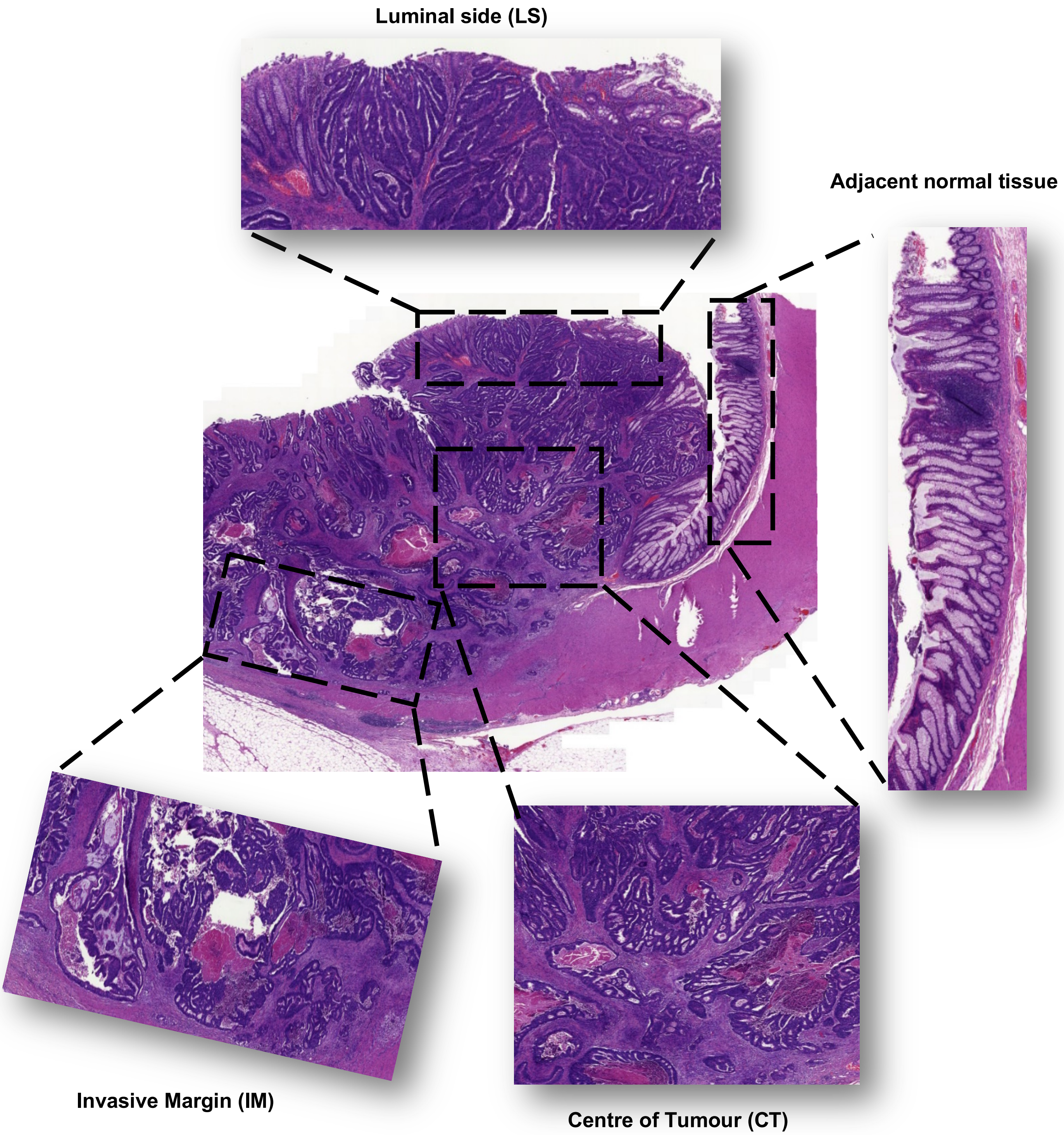

Supplementary figure 4: TMA construction consisted of three Colorectal tumour regions: Luminal side of the Tumour (LS) selected from the nearest area to the lumen; Centre of the Tumour (CT) selected from area in the middle area between the lumen and the edge of the tumour; Invasive Margin (IM) selected from the deepest area of the tumour, and adjacent normal tissue were collected from each case.

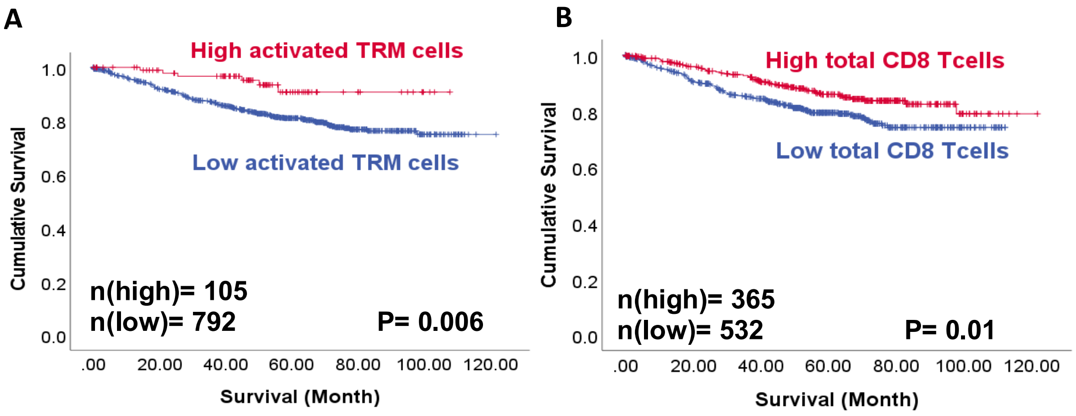

Supplementary figure 5: Prognostic impact of stromal activated T_{RM}(CD8+CD103+CD39+) and CD8 T cells in CRC patients. Kaplan-Meier plots represent the probability of disease-specific survival for **A.** activated T_{RM} and **B.** total CD8+ T cells in the stroma. Cutoff points used to stratify CRC patients into high and low-density groups were determined using X-tile. The cutoff point for total CD8+ T cells was 148 cell/mm^2 and for activated TRM cells was 52 cell/mm^2. The log-rank test was used to compare curves, and p values <0.05 were considered statistically significant.

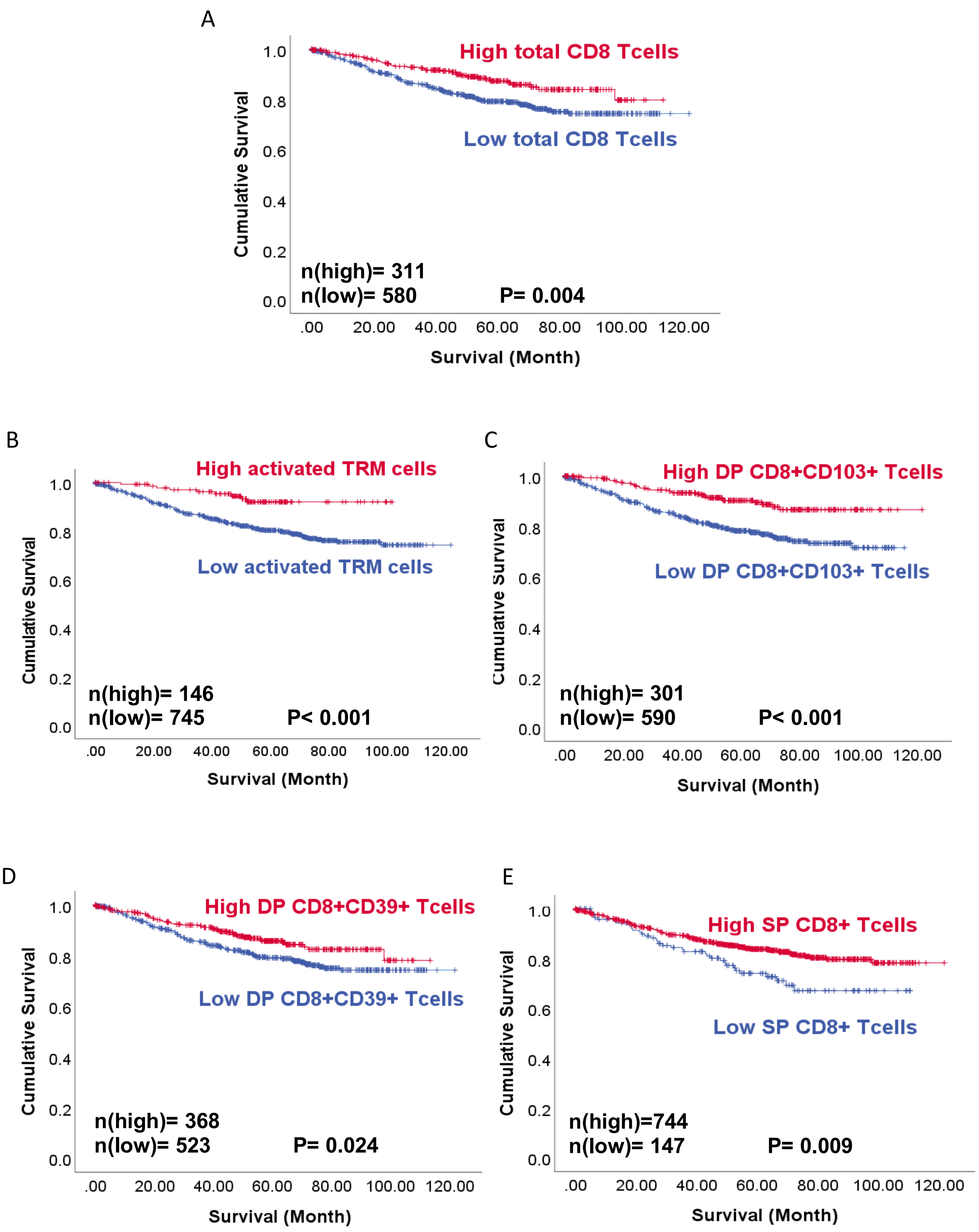

Supplementary figure 6: The effects of intraepithelial CD8 subsets infiltration on disease specific survival in CRC patients

Kaplan-Meier curves for: **(**A) intraepithelial total CD8+ T cells, (B) intraepithelial activated TRM (CD8+CD103+CD39+) T-cells, (C) intraepithelial CD8+CD103+CD39- T cells, (D) intraepithelial CD8+CD39+CD103-T cells and (E) intraepithelial CD8+CD103-CD39- T cells in the tumour epithelium of CRC patients. p values <0.05 were considered statistically significant. DP double positive; SP single positive

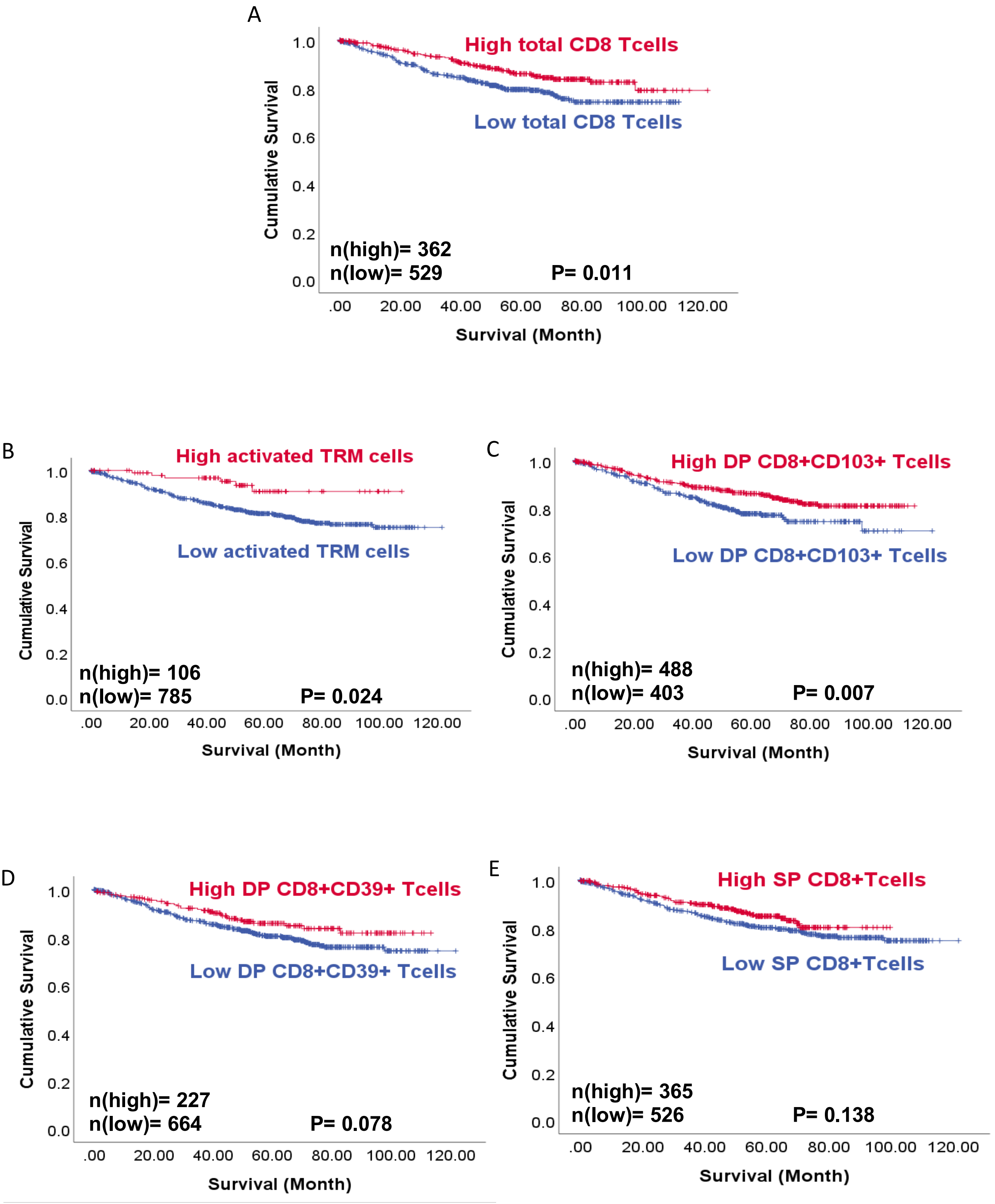

Supplementary Figure 7: The effects of stromal CD8 subsets infiltration on disease specific survival in CRC patients Kaplan-Meier Curves for disease specific survival for: (A) stromal total CD8+ T cells, (B) stromal activated T_{RM} (CD8+CD103+CD39+) T cells, (C) stromal CD8+CD103+CD39- T cells, (D) stromal CD8+CD39+CD103-T cells and (E) stromal CD8+CD103-CD39- T cells.p value <0.05 were considered statistically significant. DP double positive; SP single positive

Supplementary table 2: Correlations between the expression of intraepithelial activated T_{RM} T-cells (CD8+CD103+CD39+), CD8+CD103+CD39- T-cells, CD8+CD39+CD103-T cells and CD8+CD103-CD39- T-cells, and clinicopathologic features in CRC
Bold indicates significant p-values (<0.05) (chi-squared)

		Intraepithelial CD8+CD103+CD39+ T-cells (activated T_{RM})			Intraepithelial CD8+CD103+ CD39- T cells			Intraepithelial CD8+CD103-CD39+ T cells			Intraepithelial CD8+CD103- CD39- T cells		
		**Low (%)**	**High (%)**	**P-value**	**Low (%)**	**High (%)**	**P-value**	**Low (%)**	**High (%)**	**P-value**	**Low (%)**	**High (%)**	**P-value**
**Gender**	Male<br>Female	424 (56.5)<br>327 (43.5)	85 (58.2)<br>61 (41.8)	0.694	342 (57.1)<br>257 (42.9)	167 (56.2)<br>130 (43.8)	0.805	311 (57.8)<br>227 (42.2)	214 (55.4)<br>172 (44.6)	0.474	71 (18.3)<br>75 (14.7)	317 (81.7)<br>434 (85.3)	0.09
**Age diagnosis**	<=69<br>>69	44 (5.9)<br>707 (94.1)	10 (6.8)<br>136 (93.2)	0.645	36 (6)<br>563 (94)	18 (6.1)<br>279 (93.9)	0.976	38 (7.1)<br>500 (92.9)	21 (5.4)<br>365 (94.6)	0.320	14 (25.9)<br>132 (15.7)	40 (74.1)<br>711 (84.3)	0.057
**Tumour site**	Right colon<br>Left colon<br>Rectal<br>unknown	327 (43.5)<br>284 (37.8)<br>111 (14.8)<br>29 (3.9)	88 (60.3)<br>42 (28.8)<br>16 (11)<br>0 (0)	**0.001**	257 (42.9)<br>227 (37.9)<br>96 (16)<br>19 (3.2)	158 (53.2)<br>98 (33)<br>31 (10.4)<br>10 (3.4)	**0.017**	234 (43.5)<br>203 (37.7)<br>82 (15.2)<br>19 (3.5)	195 (50.5)<br>130 (33.7)<br>51 (13.2)<br>10 (2.6)	0.196	59 (14.2)<br>63 (19.3)	356 (85.8)<br>263 (80.7)	0.132
**Tumour grade (differentiation)**	well<br>Moderate<br>poor	13 (1.7)<br>674 (89.9)<br>63 (8.4)	4 (2.7)<br>131 (89.7)<br>11 (7.5)	0.683	7 (1.2)<br>551 (92.1)<br>40 (6.7)	10 (3.4)<br>253 (85.2)<br>34 (11.4)	**0.003**	12 (2.2)<br>484(90)<br>42 (7.8)	5 (1.3)<br>344 (89.4)<br>36 (9.4)	0.427	1 (5.9)<br>141(17.5)<br>4 (5.4)	16 (94.1)<br>664 (82.5)<br>70 (94.6)	**0.013**
**Microsatellite instability status**	Negative<br>Positive	631 (85)<br>111 (15)	109 (75.7)<br>35 (24.3)	**0.006**	518 (87.2)<br>76 (12.8)	221 (75.9)<br>70 (24.1)	**<0.001**	465 (87.2)<br>68 (12.8)	297 (78.4)<br>82 (21.6)	**<0.001**	139(18.8)<br>7 (4.8)	601 (81.2)<br>139 (95.2)	**<0.001**
**Vascular invasion**	Absent<br>Present	370 (50.1)<br>369 (49.9)	84 (58.3)<br>60 (41.7)	0.006	279 (47.4)<br>310 (52.6)	175 (59.7)<br>118 (40.3)	0.001	251 (46.9)<br>284 (53.1)	214 (56.9)<br>162 (43.1)	0.003	61 (13.4)<br>84 (19.6)	393 (86.6)<br>345 (80.4)	0.014
**TNM stage**	1<br>2<br>3<br>4	118 (15.7)<br>305 (40.6)<br>238 (31.7)<br>90 (12)	30 (20.5)<br>62 (42.5)<br>49 (33.6)<br>5 (3.4)	**0.015**	84 (14)<br>241 (40.2)<br>202 (33.7)<br>72 (12)	64 (21.5)<br>125 (42.1)<br>85 (28.6)<br>23 (7.7)	**0.007**	76 (14.1)<br>213 (39.6)<br>176 (32.7)<br>73 (13.6)	73 (18.9)<br>164 (42.5)<br>119 (30.8)<br>30 (7.8)	**0.014**	18 (12.2)<br>41 (11.2)<br>60 (20.9)<br>27 (28.4)	130 (87.8)<br>326 (88.8)<br>227 (79.1)<br>68 (71.6)	**<0.001**
**Metastases (M)**	M1 (Distant metastasis)<br>M0 (no distant metastasis)	660 (87.9)<br>91 (12.1)	141 (96.6)<br>5 (3.4)	**0.002**	526 (87.8)<br>73 (12.2)	274 (92.3)<br>23 (7.7)	**0.043**	464 (86.2)<br>74 (13.8)	356 (92.2)<br>30 (7.8)	**0.005**	119 (14.9)<br>27 (28.1)	682 (85.1)<br>69 (71.9)	**0.002**

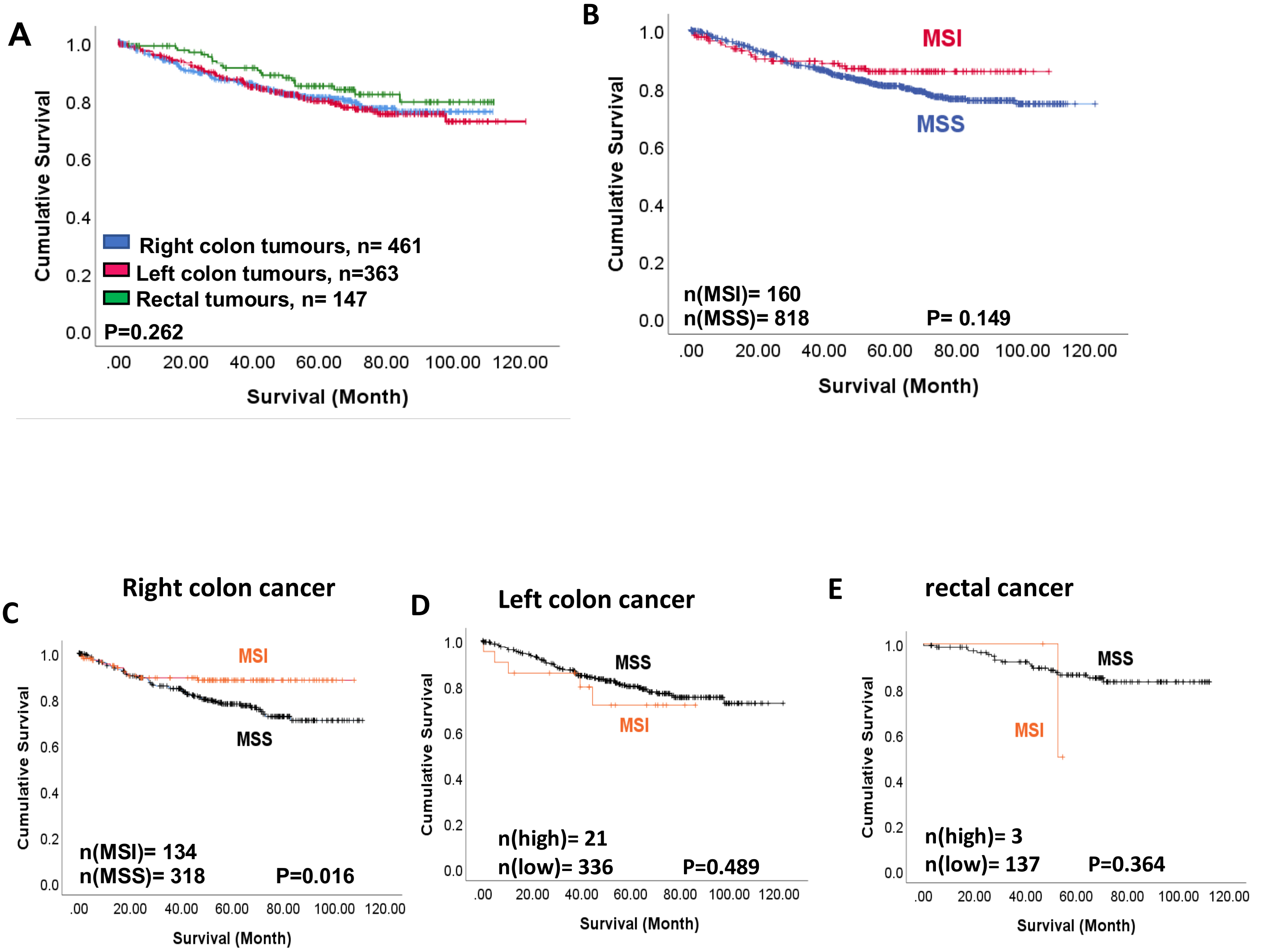

Supplementary figure 8: Kaplan Meier plots for disease-specific survival for **(A)** patients with right-sided colon cancer, left-sided colon cancer and rectal cancers**;** **(B)** patients with MSI and MSS colorectal tumours; and patients with MSI and MSS tumours in the **(C)** right colon cancer; **(D)** left colon and **(E)** rectal cancers. Log-rank test was used to test the statistical significance. p value <0.05 were considered statistically significant

	No genes	Genes up-regulated	Genes down-regulated	No. Pseudo-genes	Up-Regulated Pseudo-genes	Down regulated pseudo genes
**Right CD8^{high} CD103^{high} vs CD8^{low} CD103^{low}**	**7272**	5853	1419	1697	1532	165
**Left CD8^{high} CD103^{high} vs CD8^{low} CD103^{low}**	**877**	584	293	78	21	57
**Right CD8^{high} CD103^{low} vs CD8^{low} CD103^{low}**	**260**	85	175	66	7	59
**Left CD8^{high} CD103^{low} vs CD8^{low} CD103^{low}**	**201**	38	163	30	2	28
**Right CD8^{low} CD103^{high} vs CD8^{low} CD103^{low}**	**392**	136	256	104	45	59
**Left CD8^{low} CD103 ^{high} vs CD8^{low} CD103^{low}**	**1291**	997	294	629	572	57

Supplementary Table 3: Differential gene expression (DEG) comparing groups of CRC patients based on CD8 and CD103 expression: Data obtained from TCGA-COAD database was sorted on site of the tumour (right- or left-sides) and high (4th quartile) or low (1st quartile) expression of CD8 and CD103. Groups were compared to CD8l^{ow}CD103 ^{low} from the tumours originating from the same side. Table shows number of DEG (differential expressed genes)

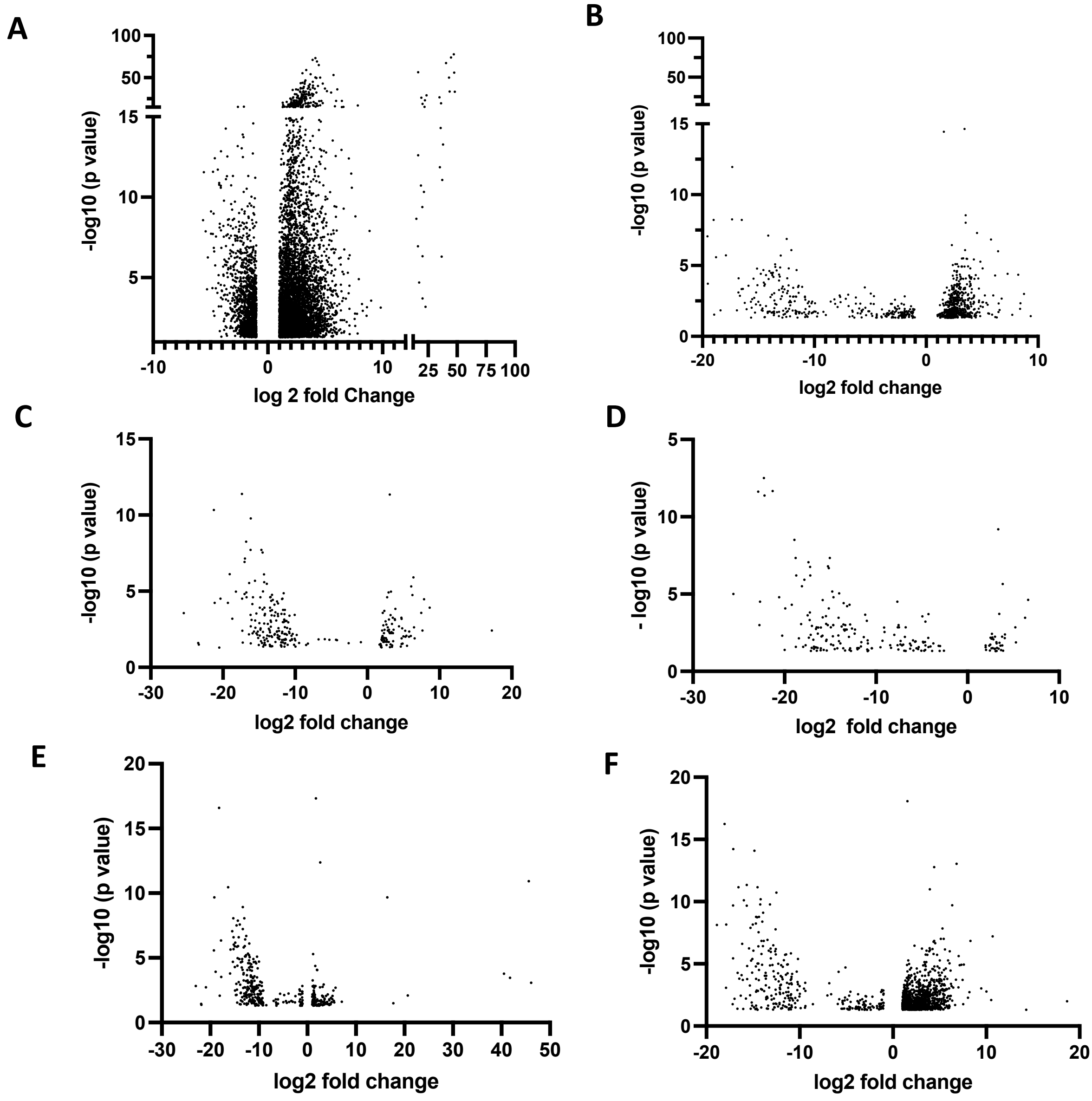

Supplementary Figure 9: Volcano plots showing the differential expressed genes (DEG)
Data obtained from TCGA-COAD database was sorted on site of the tumour (right- or left-sides) and high (4th quartile) or low (1st quartile) expression of CD8 and CD103. Groups were compared to $CD8^{low}CD103^{low}$ from the tumours originating from the same side with a positive log2 fold change represent upregulation of DEG in comparison to $CD8^{low}$ $CD103^{low}$. Only data with a log fold 2 of greater or equal to 1 and a P value of <0.05 are plotted. **A**. Right sided CD8 ^{high} $CD103^{high}$ **B.**Left-sided $CD8^{high}$ $CD103^{high}$ **C.** Right-sided $CD8^{high}$ $CD103^{low}$ **D.** Left-sided $CD8^{high}$ $CD103^{low}$ **E.** Right sided $CD8^{low}$ $CD103^{high}$ **F.** Left-sided $CD8^{low}$ $CD103^{high}$

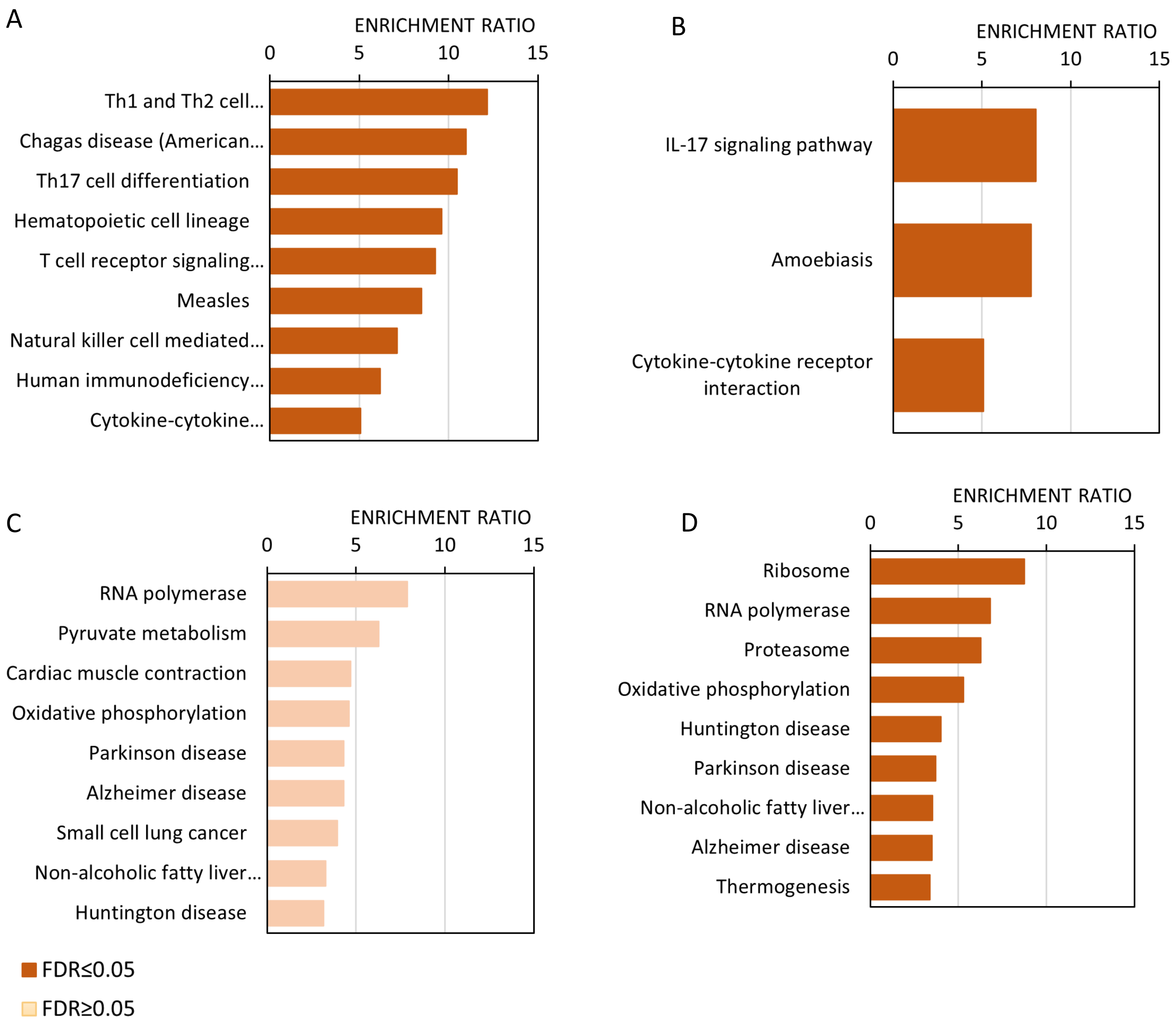

Supplementary figure 10: Webgestalt pathway analysis Data obtained from TCGA-COAD database was sorted on site of the tumour (right- or left-sides) and high (4th quartile) or low (1st quartile) expression of CD8 and CD103. Groups were compared to CD8^{low}CD103^{low} from the tumours originating from the same side. Values of greater or equal to 1 for log2 fold change and p<0.05 were analysed in the webgestalt website for pathway analysis: **(A)** right-sided CRC CD8^{high} CD103^{low} **(B)** left-sided CRC CD8^{high} CD103^{low} **(C)** right-sided CRC CD8^{low} CD103^{high} **(D)** left-sided CRC CD8^{low} CD103^{high}

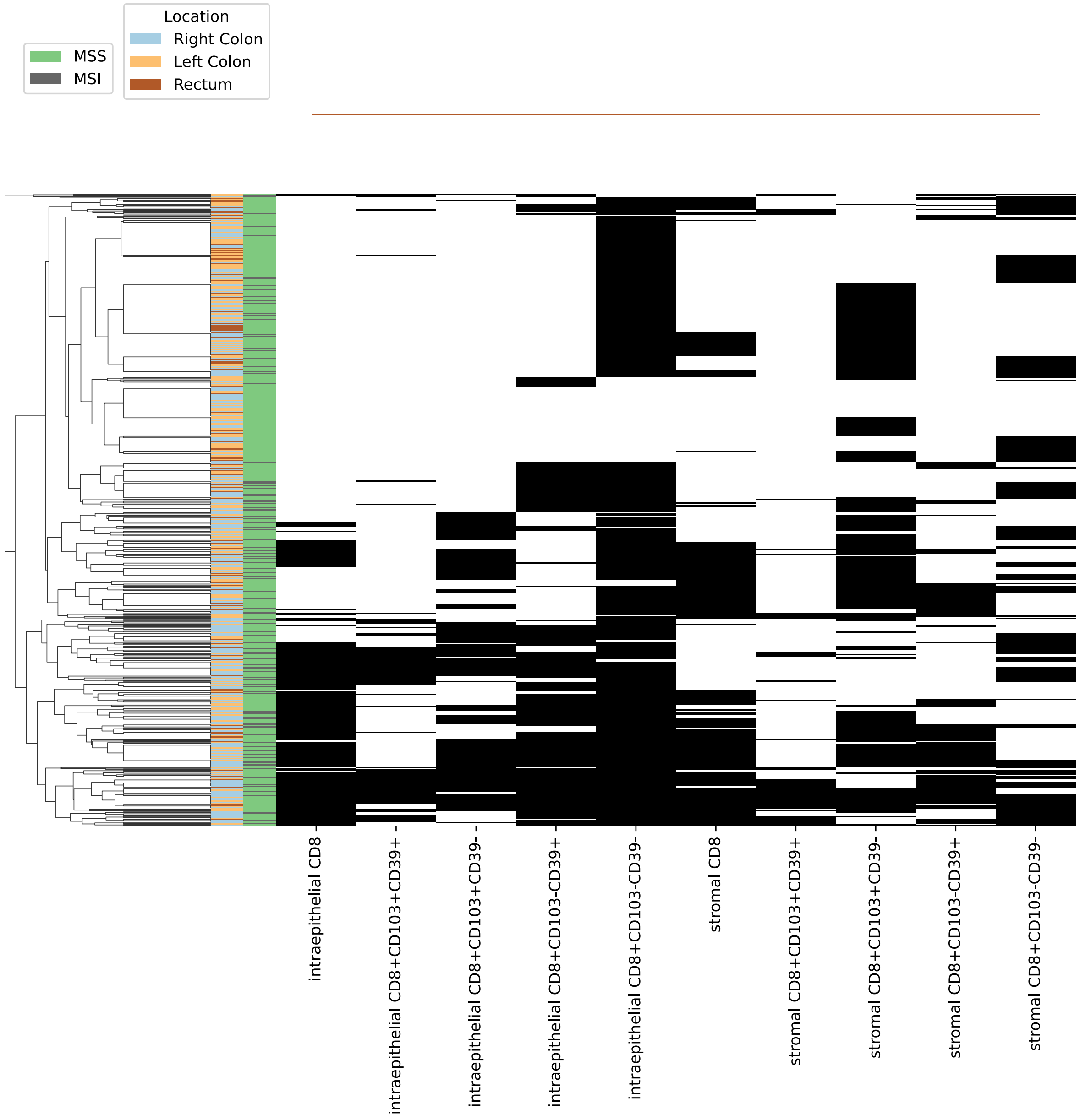

Supplementary figure 11: Heat map of High and low infiltration of each CD8+ phenotype in the epithelium and the stroma: Heat map depicting x-tile cut-off for high or low expression of each CD8 phenotype in both the epithelium and the stroma. Location of tumour is represented in left hand column with the subsequent column representing MSI and MSS status. High cell infiltration is show on the heat map as black with low depicted as white.
